# COVID-19 triggers pulmonary tumor outgrowth through hypoxia- and inflammation-induced VEGF-dependent angiogenesis

**DOI:** 10.1038/s41392-026-02952-6

**Published:** 2026-10-12

**Authors:** Xu Jing, Mingwei Ma, Yan Bai, Yaping Wu, Xiaoting Sun, Jing Wu, Kristian Holgersson, Xiaolu Wang, Lijuan Chen, Siwen Long, Gerald M. McInerney, Mikael Adner, Laszlo Szekely, Yunjian Zhang, Meiyun Wang, Jiaojiao Pang, Yuguo Chen, Yihai Cao

**Affiliations:** 1 Department of Microbiology, Tumor and Cell Biology, Karolinska Institute, Stockholm, Sweden; 2 Department of Breast Surgery, The First Affiliated Hospital of Sun Yat-sen University, Guangzhou, China; 3 Department of Radiology, Henan Provincial People’s Hospital & State Key Laboratory of Metabolic Dysregulation & Prevention and Treatment of Esophageal Cancer, Zhengzhou University, Zhengzhou, Henan China; 4 Oujiang Laboratory (Zhejiang Lab for Regenerative Medicine, Vision and Brain Health), School of Pharmaceutical Science, Wenzhou Medical University, Wenzhou, China; 5 Department of Pharmacy, First Affiliated Hospital of Shandong First Medical University and Shandong Provincial Qianfoshan Hospital, Jinan, Shandong Province China; 6 Loma Linda University, School of Medicine, Department of Basic Sciences, Loma Linda, CA USA; 7 Experimental Asthma and Allergy Research Unit, Institute of Environmental Medicine (IMM), Karolinska Institutet, Stockholm, Sweden; 8 Department of Pathology and Cytology, Karolinska University Laboratory, Stockholm, Sweden; 9 Department of Emergency Medicine, Shandong Provincial Clinical Research Center for Emergency and Critical Care Medicine, Institute of Emergency and Critical Care Medicine of Shandong University, Qilu Hospital of Shandong University, Jinan, Shandong China

**Keywords:** Oncology, Cancer

## Abstract

Pulmonary nodules are highly prevalent in adult human populations, with a substantial proportion representing dormant cancerous lesions. We hypothesized that these pulmonary dormant cancers debilitate in growth owing to the lack of an angiogenic switch in tumors. In this study, we comprehensively investigated the hypothesis that specific pathological triggers can activate this angiogenic switch utilizing severe acute respiratory coronavirus 2 (SARS-CoV-2) infection-induced hypoxia as a primary physiological stimulus. Our findings provide compelling experimental evidence demonstrating that SARS-CoV-2 infection induces profound pulmonary hypoxia and severe tissue inflammation in animal models. This microenvironmental disruption markedly upregulates the expression of vascular endothelial growth factor (VEGF) and vascular endothelial growth factor receptor 2 (VEGFR2) within lung tissues. The resulting hyperactivation of the downstream VEGF-VEGFR2 signaling pathway effectively drives robust pulmonary neovascularization, a phenomenon frequently documented in clinical observations of COVID-19 patients. Furthermore, employing an established experimental tumor model, we demonstrated that SARS-CoV-2 infection directly accelerates tumor outgrowth via this specific VEGF angiogenesis-dependent cascade. Crucially, the targeted administration of a neutralizing antibody to block VEGF significantly suppressed tumor progression and outgrowth in SARS-CoV-2-infected, tumor-bearing animals. Our data show that the COVID-19-induced hypoxia-VEGF-angiogenesis axis serves as one of the mechanisms for initiating tumor outgrowth in pulmonary tissues.

## Introduction

Clinical tumor dormancy is defined as the growth arrest of tumors in their primary sites or metastatic locations.^1,2^ Dormant tumors often exist in small sizes in the affected tissues and organs for months, years, or even decades without growth.^3–5^ Microscopically dormant tumors emerge as a cluster of tumor cells that can only be detected under histological examinations.^6^ Several mechanisms have been proposed to induce tumor dormancy, including escape from immune surveillance; incapacitating tumor angiogenesis; lack of appropriate cell matrix to support tumor growth; unfavorable tumor microenvironment (TME) to support tumor growth; metabolic reprogramming in cancer cells; and cancer cell cycle arrest.^7–11^ Each of these mechanisms may alone or cooperatively contribute to tumor dormancy.

In experimental animal tumor models, tumors remain avascular dormancy in their tiny sizes due to the lack of an angiogenic phenotype.^12–14^ However, malignant cells in a dormant tumor seem to represent a tightly regulated balance between cell proliferation and cell death.^2,15^ Removal of angiogenesis suppression triggers the outgrowth of dormant tumors,^14,16^ indicating the essential role of switching to an angiogenic phenotype in supporting tumor growth.^17^ Tumors produce multifarious angiogenic factors to support their growth.^18^ Among various tumor-derived proangiogenic factors, vascular endothelial growth factor (VEGF) is probably the most extensively studied angiogenic factor in various tumors.^18–20^ The expression levels of VEGF are often increased in most tumors owing to genetic, epigenetic, and microenvironmental factors, including but not limited to oncogenic mutations, DNA methylation, hypoxia, inflammation, and fibrosis.^19,21^ VEGF displays multiple functions mainly through the VEGFR2 receptor, although vascular endothelial growth factor receptor 1 (VEGFR1) and neuropilin receptors may also participate in VEGF-mediated signaling activation.^18,19^ In addition to stimulating angiogenesis, other vascular functions of VEGF include increasing vascular permeability, vascular remodeling, and endothelial cell survival.^19,22^ Local tumor-derived and systemic VEGF molecules are both involved in executing these vascular functions in tumors.^18,23^

The SARS-CoV-2 virus primarily infects epithelial cells in the lungs and airways, which in severe cases causes dyspnea owing to extensive inflammation, angiogenesis, plasma extravasation into the alveolar space, and fibrosis.^24,25^ Coronavirus disease 2019-triggered (COVID-19) dyspnea not only induces hypoxia in local pulmonary tissues but also triggers systemic hypoxia in all organs and tissues owing to inefficient oxygen exchange in the lungs.^26^ Hypoxia often provokes numerous pathological responses, such as angiogenesis, vascular dilation, inflammation, cell death, and tissue necrosis.^27^ In response to hypoxia, VEGF is upregulated through transcriptional activation by hypoxia-inducible factor-1 (HIF-1).^28–30^ VEGF significantly contributes to the abovementioned pathological processes in response to SARS-CoV-2 infection.^31^ Consequently, anti-VEGF therapy demonstrated clinical benefits for effective treatment of severe COVID-19 in human patients.^31,32^ Several clinical studies based on large human cohorts demonstrated positive correlations of COVID-19 and high prevalences of lung nodules and cancers.^33–36^ For example, a comparison of prepandemic and postpandemic groups of 18,906 chest computed tomography (CT) scans demonstrated a marked increase in lung nodules in the postpandemic group.^37^ Similarly, analysis of 112,311 individuals in the COVID-19 group showed increases in lung cancer risks.^38^ However, the molecular mechanisms of the COVID-19-associated high risks of lung nodules and cancers remain largely unknown.

In this study, we proposed a new hypothesis that COVID-19-augmented VEGF expression succors to tumor outgrowth by switching to an angiogenic phenotype that initiates tumor outgrowth. We provide compelling experimental evidence to show that the SARS-CoV-2 infection-initiated tumor growth of lung nodules is dependent on VEGF-induced angiogenesis. Blocking VEGF with a neutralizing antibody (VEGF blockade) repressed tumor outgrowth in SARS-CoV-2-infected tumor-bearing mice. We believe that these findings may imply potentially high incidences of clinically manifested lung cancers by other pulmonary infectious diseases and hypoxic insults.

## Results

### Neovascularization, elevated VEGF production and inflammation in COVID-19 lung tissues

To recapitulate clinical COVID-19 in human patients, we developed a clinically relevant COVID-19 mouse model in which the original strain of SARS-CoV-2 virus was used to infect immunocompetent heterozygous K18-hACE C57BL/6 J mice.^39^ All these experiments were performed in a P3 laboratory under the strict regulations of the Karolinska Institutet and the ethical regulations of the Stockholm Ethical Committee. Each of the 10-week-old male heterozygous K18-hACE C57BL/6 J mice received 100 PFU of SARS-CoV-2 virus through intranasal delivery using our previously published protocols.^39^ On day 7 postinfection, the mice were sacrificed, and the relevant tissues and organs were collected for their respective analyses.

**Fig. 1:**
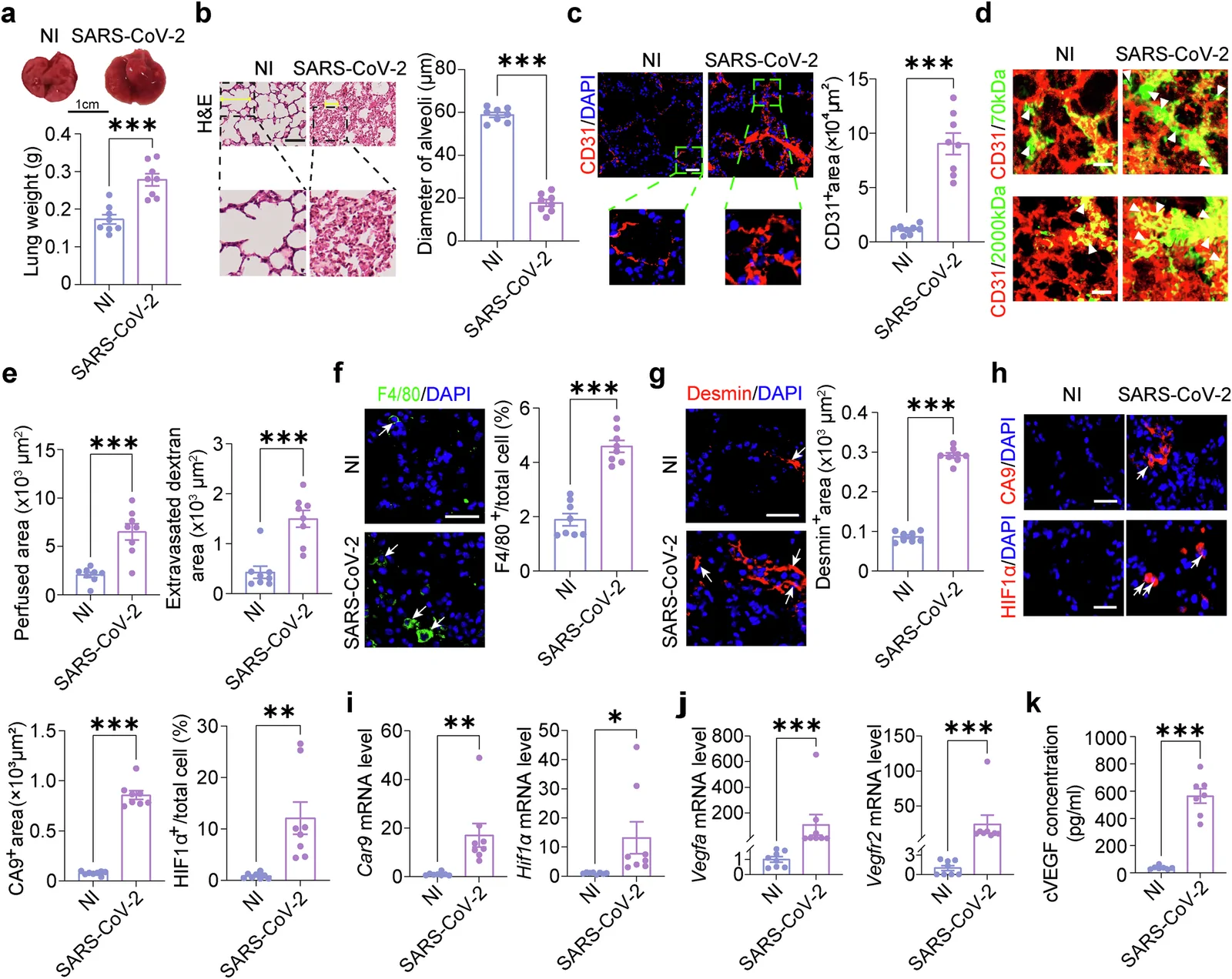
SARS-CoV-2 infection in a murine model. **a** Representative photographs of lungs from noninfected (NI) and SARS-CoV-2-infected mice. Quantification of lung weight (n = 8 mice per group). **b** Representative H&E-stained lung sections from NI and SC mice. Quantification of the average alveolar diameter (*n* = 8 fields per group). **c** Representative immunofluorescence staining for CD31 (red) in lung sections. Nuclei are counterstained with DAPI (blue). Quantification of the CD31-positive area (*n* = 8 fields per group). **d** Representative fluorescence images showing LRD distribution (green) and CD31-stained vasculature (red) in lungs Extravascular leakage of 70 kDa LRD indicates vascular permeability, whereas 2,000 kDa LRD reflects vascular perfusion. Arrowheads indicate regions of vascular perfusion (yellow) or dextran extravasation (green). **e** Quantification of the LRD-extravasated (green) area and perfused vessel (yellow) area (*n* = 8 fields per group). **f** Representative immunofluorescence staining for F4/80 (green) in lung sections. Nuclei are counterstained with DAPI (blue). Quantification of the ratio of F4/80-positive cells to total cells (*n* = 8 fields per group). **g** Representative immunofluorescence staining for desmin (red) in lung sections. Nuclei are counterstained with DAPI (blue). Quantification of the Desmin-positive area (*n* = 8 fields per group). **h** Representative immunofluorescence staining for HIF1α (red) and CA9 (red) in lung sections. Nuclei are counterstained with DAPI (blue). Quantification of the ratio of HIF1α-positive cells and the CA9-positive area (*n* = 8 fields per group). **i** Relative mRNA expression levels of Car9 and Hif1α in lung tissues, as determined by qPCR (*n* = 8 samples per group). **j** Relative mRNA expression levels of Vegf and Vegfr2 in lung tissues, as determined by qPCR (*n* = 8 samples per group). **k** Concentration of VEGF protein in lung tissue homogenates, measured by ELISA (*n* = 7 samples per group). Data are presented as the mean ± s.e.m. *p < 0.05, **p < 0.01, ***p < 0.001; two-sided unpaired t test. Scale bars = 50 μm

Gross examination of lung tissues showed increased shadier color in the SARS-CoV-2-infected lungs relative to the noninfected controls (Fig. 1a). The total tissue weight of the SARS-CoV-2-infected lungs was also significantly increased (Fig. 1a). Hematoxylin & eosin (H&E) staining of lung tissues demonstrated marked increases in cellular components in SARS-CoV-2-infected lungs, resulting in decreased diameters of the pulmonary alveolus (Fig. 1b). Extravasation of plasma fluid together with other cellular components was frequently found in the alveolar space (Fig. 1b). One of the distinguished features was the markedly increased density of CD31^+^ microvessels in SARS-CoV-2-infected lung tissues (Fig. 1c). Quantification analysis showed an over 9-fold increase in CD31^+^ structures compared with the noninfected controls (Fig. 1c). Microvessels in the SARS-CoV-2-infected lung tissues showed increased perfusion and permeability relative to the noninfected controls, as detected by rhodamine-labeled 2000-kD and 70-kD dextran, respectively (Fig. 1d).

In addition to hyperneovascularization, F4/80^+^ macrophages and desmin^+^ fibrotic components were also markedly increased in SARS-CoV-2-infected lung tissues (Fig. 1f, g). Both CD80^+^ M1-like and CD206^+^ M2-like macrophages were increased in SARS-CoV-2-infected lungs (Suppl. Fig. 1). Notably, the total CD3^+^ T-cell population, CD4^+^ T cells, and CD8^+^ T cells significantly decreased (Suppl. Fig. 2), reconciling COVID-19-triggered lymphopenia.^40^ Additionally, FAP^+^ fibroblasts and αSMA^+^ myofibroblasts were increased (Suppl. Fig. 3). Ki67^+^ proliferating desmin+ fibroblasts and TUNEL^+^ apoptotic fibroblasts were increased and represented a new balance under SARS-CoV-2 infection (Suppl. Fig. 4a). A substantial number of CD3^+^ T cells, CD4^+^ and CD8^+^ and F4/80^+^ macrophages underwent apoptosis without proliferation (Suppl. Fig. 4 and 5).

These COVID-19-associated pathological changes markedly altered the pulmonary microenvironment and resulted in severe hypoxia. Probing CA9 demonstrated that severe hypoxia existed in the SARS-CoV-2-infected lungs versus those of controls (Fig. 1h, i). Consistently, the expression levels of HIF-1α mRNA and protein were also markedly increased (Fig. 1h, i). Since VEGF expression is regulated by tissue hypoxia, we next measured VEGF expression in SARS-CoV-2-infected lung tissues. Intriguingly, an approximately 180-fold increase in Vegf mRNA was detected in SARS-CoV-2-infected lung tissues relative to that of noninfected controls (Fig. 1j). Measurement of circulating VEGF levels in the peripheral blood showed a more than 3-fold increase in SARS-CoV-2-infected animals (Fig. 1k). Finally, VEGFR2, as a functional receptor, was also markedly upregulated by approximately 50-fold (Fig. 1j). Interestingly, VEGFR1, as a decoy receptor for VEGF signaling, lacked significant changes between the two groups (Suppl. Fig. 4b). Upregulation of VEGFR2 further enhances VEGF signaling. Together, SARS-CoV-2 infection results in exceptionally high expression of VEGF and VEGFR2, which potentially augment angiogenesis in lung tissues.

### SARS-CoV-2 infection stimulates lung tumor outgrowth

**Fig. 2:**
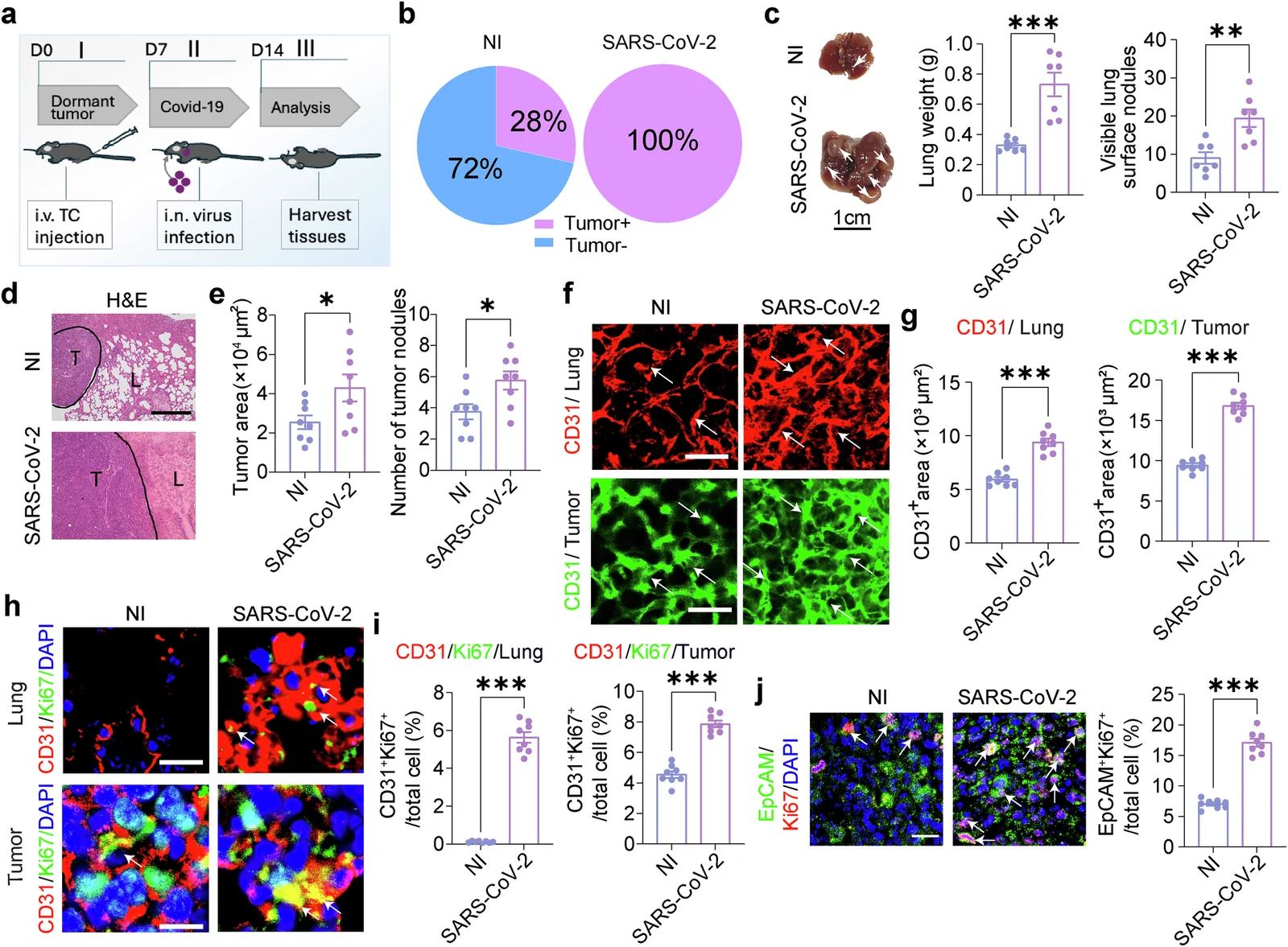
SARS-CoV-2 infection in lung tumor development and progression. **a** Experimental timeline showing intravenous injection of Lewis lung carcinoma cells, SARS-CoV-2 infection, and tissue harvest schedule. **b** Tumor formation rates across experimental groups. **c** Representative lung images from each group. Lung weight quantification and enumeration of surface tumor nodules (*n* = 7 mice per group). **d** H&E-stained lung sections showing tumor foci and adjacent pulmonary architecture. **e** Measurements of individual tumor size and tumor area per lung section (*n* = 8 fields per group). **f** Immunofluorescence staining of CD31 showing vascular patterns in tumor regions (green) and versus adjacent lung tissue (red); arrows indicate regions of neovascular structures. **g** Quantification of CD31-positive area in tumor regions and versus adjacent lung tissue (*n* = 8 fields per group). **h** Immunofluorescence costaining of CD31 (red) and Ki67 (green) demonstrating proliferating endothelial cells in separate panels for tumor regions and adjacent lung tissue. Nuclei are stained with DAPI (blue). **i** Quantification of CD31 and Ki67 double-positive cells in tumor regions and versus adjacent lung tissue (*n* = 8 fields per group). **j** Immunofluorescence costaining of Ki67 (red) and EpCAM (green) demonstrating proliferating tumor cells. Nuclei are stained with DAPI (blue). Quantification of Ki67 and EpCAM double-positive cells in tumor regions (*n* = 8 fields per group). Data are presented as the mean ± s.e.m. Statistical analysis was performed using two-sided unpaired t tests. *p < 0.05, **p < 0.01, ***p < 0.001. Scale bars = 50 μm

Because SARS-CoV-2 infection markedly increased pulmonary VEGF and VEGFR2 expression levels and microvascular density, we hypothesized that COVID-19-induced angiogenesis might promote tumor outgrowth. Pulmonary cancerous nodules might remain in their dormant stage for months and years without further growth because of a lack of sufficient proangiogenic factors to switch to an angiogenic phenotype.^14–16,41^ SARS-CoV-2 infection-induced high VEGF expression potentially contributes to an angiogenic switch that promotes tumor outgrowth. To test this hypothesis, we developed a dormant lung cancer model in heterozygous K18-hACE C57BL/6 J immunocompetent mice that were subsequently infected by SARS-CoV-2 virus (Fig. 2a). In non-SARS-CoV-2-infected control animals, only a minority of mice (28%) developed lung cancers after intravenous injection of 1 × 10^6^ Lewis lung carcinoma (LLC) tumor cells per animal (Fig. 2b). In contrast, 100% of COVID-19 mice developed visible tumors on the surface of the lungs after injection of the same number of LLC tumor cells (Fig. 2b, c). H&E histology confirmed the existence of lung cancers (Fig. 2d). Again, the number of tumor lesions and the average size of tumors were significantly increased in the SARS-CoV-2-infected group (Fig. 2e).

As expected, the numbers of CD31^+^ microvessels in the SARS-CoV-2-infected lung tissues were markedly increased relative to those in noninfected lung tissues (Fig. 2f, g). Similarly, tumor CD31^+^ microvessels were also markedly increased in the COVID-19 group (Fig. 2f, g). To provide definitive evidence of COVID-19-triggered pulmonary angiogenesis and tumor angiogenesis, we performed double anti-CD31 and Ki67 immunostaining of lung tissues and tumor tissues. Indeed, intranasal SARS-CoV-2 infection instigated an angiogenic phenotype of high proliferation of Ki67^+^/CD31^+^ endothelial cells (Fig. 2h, i). Furthermore, double immunostaining of tumor tissues with a tumor cell-specific epithelial cell adhesion molecule (EpCAM) marker and Ki67 defined high proliferation of tumor cells in the SARS-CoV-2-infected animals relative to those tumors grown in the noninfected control animals (Fig. 2j). The proliferating Ki67^+^/CD31^+^ endothelial cells only existed in the SARS-CoV-2-infected lung tissues but not in the noninfected control lung tissues (Fig. 2h, i). Interestingly, high endothelial cell proliferation was balanced with increased apoptosis, demonstrating high turnover of endothelial cells (Suppl. Fig. 4). High numbers of Ki67^+^/CD31^+^ double-positive signals were also detected in LLC tumors growing in SARS-CoV-2-infected animals relative to the control animals (Fig. 2h, i). Measurement of circulating VEGF levels in the peripheral blood showed a more than 10-fold increase in SARS-CoV-2-infected animals (Suppl. Fig. 6a), further supporting a systemic pro-angiogenic response. These data demonstrate that SARS-CoV-2 triggers an angiogenic phenotype in lung tissues, which instigates tumor outgrowth. Consistently, tumor tissues from SARS-CoV-2-infected mice exhibited increased expression of CA9 and HIF1α, accompanied by enhanced Ki67^+^ proliferation and TUNEL^+^ apoptosis, reflecting a hypoxic and metabolically active microenvironment (Suppl. Fig. 6b). Moreover, CD3^+^, CD4^+^, and CD8^+^ T cells were markedly reduced, indicating COVID-19-associated tumor immune suppression (Suppl. Fig. 7). Consistently, increased TUNEL^+^ apoptosis was detected in the SARS-CoV-2-infected lung tissues relative to the noninfected controls (Suppl. Fig. 4). Both F4/80^+^ macrophages with M1-like (CD80^+^, CD86^+^) and M2-like (CD163^+^, CD206^+^) phenotypes were increased (Suppl. Fig. 8). In addition, FAP^+^, desmin^+^, and αSMA^+^ stromal fibroblasts and myofibroblasts were expanded, suggesting active tumor-associated fibrosis under SARS-CoV-2 infection (Suppl. Fig. 9). These data show that the COVID-19-altered pulmonary microenvironment with hyperneovascularization facilitates dormant outgrowth.

### VEGF-dependent tumor outgrowth

**Fig. 3:**
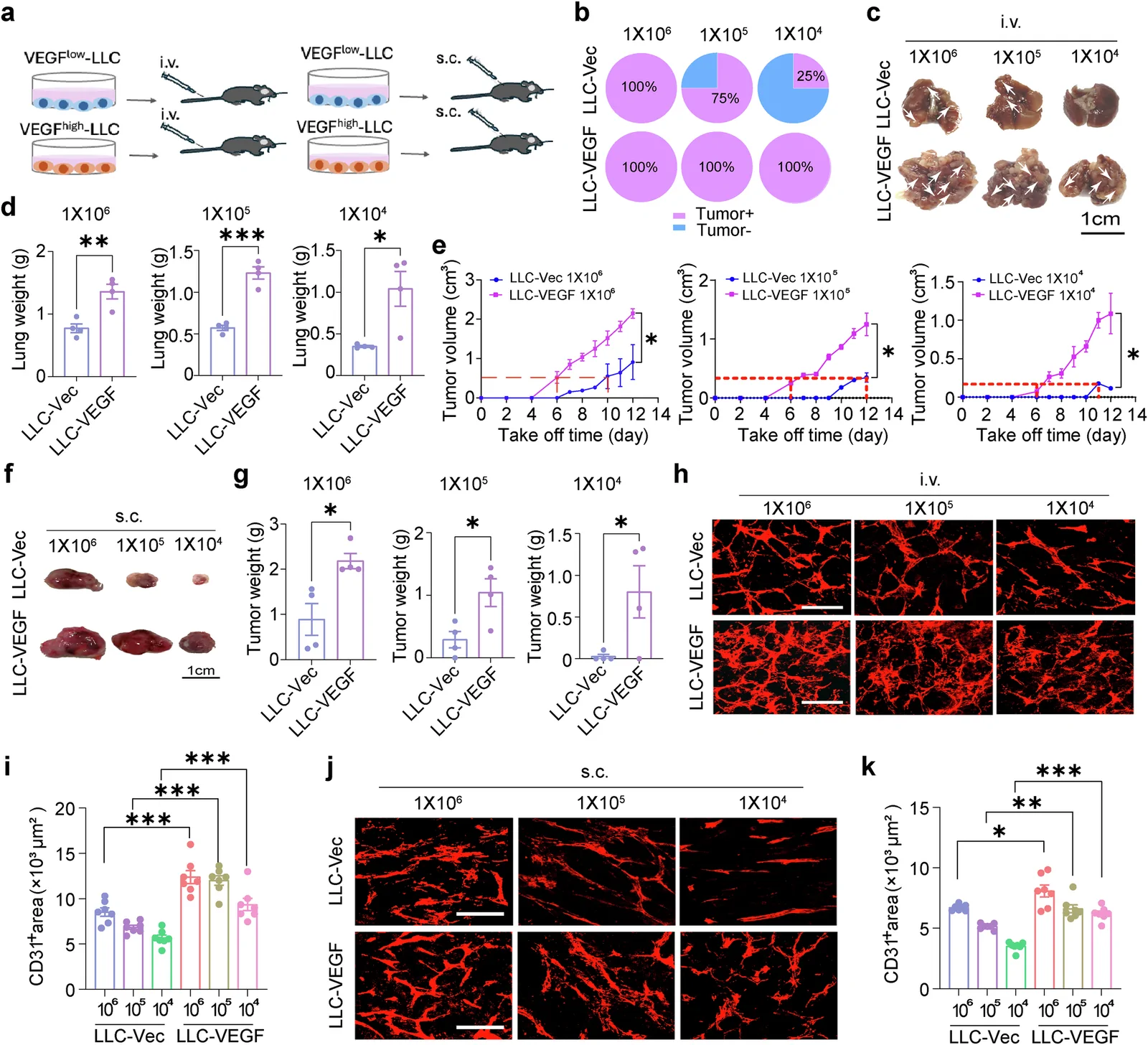
VEGF overexpression promotes tumor growth. **a** Experimental timeline depicting intravenous (i.v.) or subcutaneous (s.c.) injection of Lewis lung carcinoma cells overexpressing VEGF (LLC-VEGF) or empty vector control (LLC-Vec). **b** Tumor formation incidence following i.v. injection at different cell doses (1 × 10^4^, 1 × 10^5^, 1 × 10^6^). **c** Representative photographs of lungs from mice receiving i.v. injection of LLC-Vec or LLC-VEGF cells. **d** Quantification of lung weight (*n* = 4 mice per group). **e** Tumor growth curves from the s.c. implantation model (*n* = 4 tumors per group). **f** Representative photographs of subcutaneous tumors from the LLC-Vec and LLC-VEGF groups. **g** Quantification of subcutaneous tumor weight (*n* = 4 tumors per group). **h** Representative immunofluorescence staining for CD31 (red) in lung tumor sections from the i.v. model. **i** Quantification of the CD31-positive area in lung tumors (*n* = 7 fields per group). **j** Representative immunofluorescence staining of CD31 (red) in subcutaneous tumor sections. **k** Quantification of the CD31-positive area in subcutaneous tumors (*n* = 7 fields per group). Data are presented as the mean ± s.e.m. *p < 0.05, **p < 0.01, ***p < 0.001; two-sided unpaired t test. Scale bars = 50 μm

On the basis of the abovementioned results, we further hypothesized that the levels of proangiogenic factors might act as key determinants for tumor formation and initiation of tumor outgrowth. To test this hypothesis, we genetically propagated the LLC lung cancer cell line to express a high level of VEGF. Quantification analysis by an enzyme-linked immunosorbent assay (ELISA) showed that after 24 h of incubation, 300 ng VEGF was produced by 2.0 × 10^5^ VEGF^high^-LLC tumor cells, a 100-fold increase in VEGF expression relative to the control VEGF^low^-LLC cells (Figure S10a). Both VEGF^low^-LLC and VEGF^high^-LLC tumor cells were subcutaneously or intravenously implanted into C57BL6/NRj mice (Fig. 3a). To investigate the role of VEGF in tumor formation, we performed a titration experiment in which tumor cell numbers were successively reduced (Fig. 3b, c). Intravenous injection of 1 × 10^6^ tumor cells into each mouse resulted in 100% tumor takeoff in lung tissues in both the VEGF^low^-LLC and VEGF^high^-LLC models (Fig. 3b). However, injection of 1 × 10^5^ cells into each mouse instigated tumor formation in 75% of VEGF^low^-LLC-implanted animals and 100% tumor formation in the VEGF^high^-LLC tumor model (Fig. 3b). A further decrease in the cell number to 1 × 10^4^ cells per mouse resulted in further polarization of tumor formation between the VEGF^low^-LLC and VEGF^high^-LLC groups (Fig. 3b). Under this experimental setting, only 25% of the VEGF^low^-LLC-implanted animals were able to form tumors, whereas 100% of the VEGF^high^-LLC-implanted mice formed detectable tumors (Fig. 3b). These data show that intravenous injection of 1 × 10^4^ control tumor cells per animal resulted in lung tumor development in only a minority of mice and that elevation of VEGF production markedly increased tumor formation by 100%. These findings demonstrate VEGF-dependent formation of lung cancers at a very low number of tumor cells. Since most clinically dormant tumors consist of a low number of tumor cells, these findings are relevant to dormant tumors in human patients.

In addition to facilitating tumor formation, high VEGF expression also accelerated the tumor growth rate of LLC tumors (Fig. 3c–g). In both subcutaneous and intravenous tumor models, VEGF^high^-LLC exhibited accelerated growth rates relative to VEGF^low^-LLC, as measured by tumor volumes, tumor burden, tumor morphology, and lung surface tumor lesions (Fig. 3c–g). Notably, in the VEGF^low^-LLC group at 1 × 10^4^ tumor cells per animal, many tumors remained in their dormant stage, which were retained in tiny sizes and had a pale appearance (Fig. 3c, d, f, g). These morphological phenotypes are typical characteristics of avascular dormant tumors.^14–16,41,42^ Consistent with accelerated tumor growth rates, VEGF^high^-LLC tumors contained an increased density of CD31^+^ microvessels compared with their relative controls (Fig. 3h, i). Similarly, high numbers of CD31^+^ microvessels in VEGF^high^-LLC tumors were also validated in a subcutaneous tumor model (Fig. 3j, k). These findings further support the VEGF-dependent mechanism of tumor formation and tumor growth as described in the COVID-19 model.

### Blocking VEGF inhibits tumor outgrowth

**Fig. 4:**
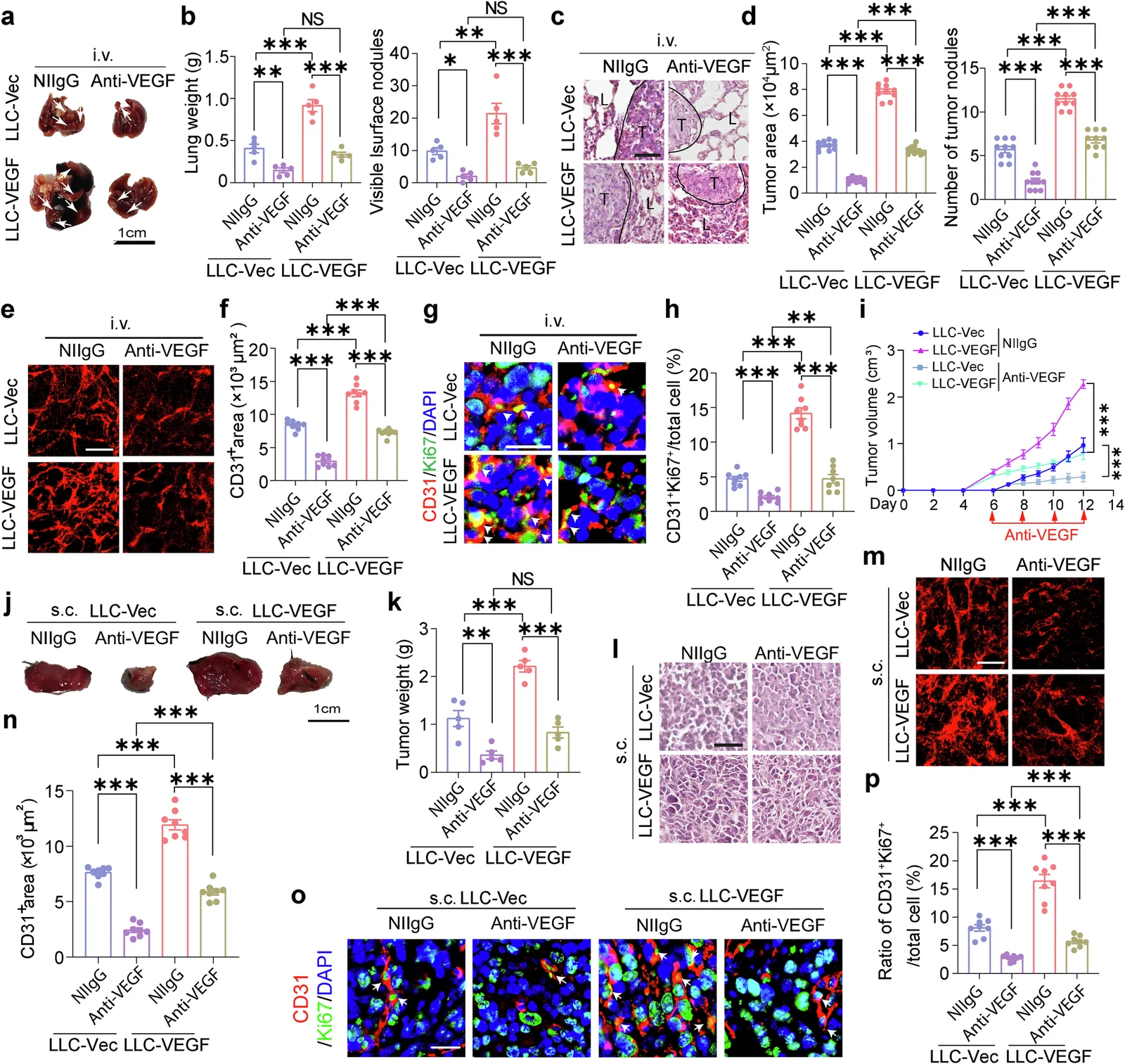
Anti-VEGF therapy inhibits tumor growth. **a** Representative lung photographs of nonimmune IgG (NIIgG)- and anti-VEGF-treated LLC-Vec- and LLC-VEGF-injected mice. **b** Quantification of lung weight and enumeration of visible surface tumor nodules in LLC-Vec- and LLC-VEGF-injected mice treated with NIIgG or anti-VEGF antibody (*n* = 5 mice per group). **c** H&E-stained lung sections of LLC-Vec- and LLC-VEGF-injected mice treated with NIIgG or anti-VEGF antibody. Tumor foci (T) and adjacent lung tissue (L) are demarcated. **d** Measurements of individual tumor size and tumor area per lung section in LLC-Vec- and LLC-VEGF-injected mice treated with NIIgG or anti-VEGF antibody (*n* = 10 fields per group). **e** Immunofluorescence staining of CD31 (red) in lung tumor sections of LLC-Vec- and LLC-VEGF-injected mice treated with NIIgG or anti-VEGF antibody. **f** Quantification of the CD31-positive area in lung tumors from LLC-Vec- and LLC-VEGF-injected mice treated with NIIgG or anti-VEGF antibody (*n* = 8 fields per group). **g** Immunofluorescence costaining of CD31 (red) and Ki67 (green) in lung tumor sections of LLC-Vec- and LLC-VEGF-injected mice treated with NIIgG or anti-VEGF antibody. Nuclei are stained with DAPI (blue). **h** Quantification of CD31 and Ki67 double-positive cells in lung tumors of LLC-Vec- and LLC-VEGF-injected mice treated with NIIgG or anti-VEGF antibody (*n* = 8 fields per group). **i** Subcutaneous tumor growth curves in LLC-Vec- and LLC-VEGF-injected mice treated with NIIgG or anti-VEGF antibody (n = 5 tumors per group). **j** Representative photographs of subcutaneous tumors of LLC-Vec- and LLC-VEGF-injected mice treated with NIIgG or anti-VEGF antibody. **k** Quantification of subcutaneous tumor weight in LLC-Vec- and LLC-VEGF-injected mice treated with NIIgG or anti-VEGF antibody (*n* = 5 tumors per group). **l** H&E staining of subcutaneous tumor sections of LLC-Vec- and LLC-VEGF-injected mice treated with NIIgG or anti-VEGF antibody. **m** Immunofluorescence staining of CD31 (red) in subcutaneous tumors of LLC-Vec- and LLC-VEGF-injected mice treated with NIIgG or anti-VEGF antibody. **n** Quantification of CD31-positive area in subcutaneous tumors of LLC-Vec- and LLC-VEGF-injected mice treated with NIIgG or anti-VEGF antibody (*n* = 8 fields per group). **o** Immunofluorescence costaining of CD31 (red) and Ki67 (green) in subcutaneous tumors of LLC-Vec- and LLC-VEGF-injected mice treated with NIIgG or anti-VEGF antibody. Nuclei are stained with DAPI (blue). **p** Quantification of CD31 and Ki67 double-positive cells in subcutaneous tumors of LLC-Vec- and LLC-VEGF-injected mice treated with NIIgG or anti-VEGF antibody (*n* = 8 fields per group). Data are presented as the mean ± s.e.m. *p < 0.05, **p < 0.01, ***p < 0.001; one-way ANOVA with Tukey’s post hoc test. NS not significant. Scale bars = 50 μm

To provide further experimental evidence of VEGF-dependent tumor formation and outgrowth, a humanized rabbit anti-VEGF antibody (VEGF blockade, Suvemcitug) that neutralizes both human and mouse VEGF was employed in our experimental settings.^43,44^ This anti-VEGF antibody has recently received China FDA approval for the treatment of metastatic ovarian cancer in human patients.^45^ In the intravenous lung cancer model, treatment with this VEGF blockade markedly reduced the number of visible tumor nodules on the surface of lung tissues (Fig. 4a, b). Quantification of the lung surface tumor nodules showed no significant differences between the anti-VEGF-treated VEGF-LLC and control LLC tumors, supporting VEGF-dependent tumor formation and growth (Fig. 4b). Treatment of tumor-bearing animals with a control IgG at the same dose had no impact on tumor formation and growth in either control or VEGF tumors (Fig. 4a, b). Notably, VEGF blockade also inhibited tumor outgrowth in the control tumors, which was in line with the general concept of the antitumor effects of anti-VEGF therapy.^18^

Histological analysis further corroborated the antitumor effects of VEGF blockade in suppressing tumor nodule formation and tumor growth (Fig. 4c, d). In line with these findings, VEGF blockade markedly decreased the microvessel density in both control and VEGF tumors (Fig. 4e, f). CD31 and Ki67 double immunostaining demonstrated a marked decrease in proliferating endothelial cells in tumor vessels in response to anti-VEGF therapy (Fig. 4g, h). These findings from the subcutaneous tumor model successfully validated our data from the intravenous tumor models (Fig. 4i–p). Together, these results demonstrate that VEGF is essentially required for tumor formation and tumor growth in our experimental settings.

### Switching to an angiogenic phenotype by COVID-19 in human lung tissues

**Fig. 5:**
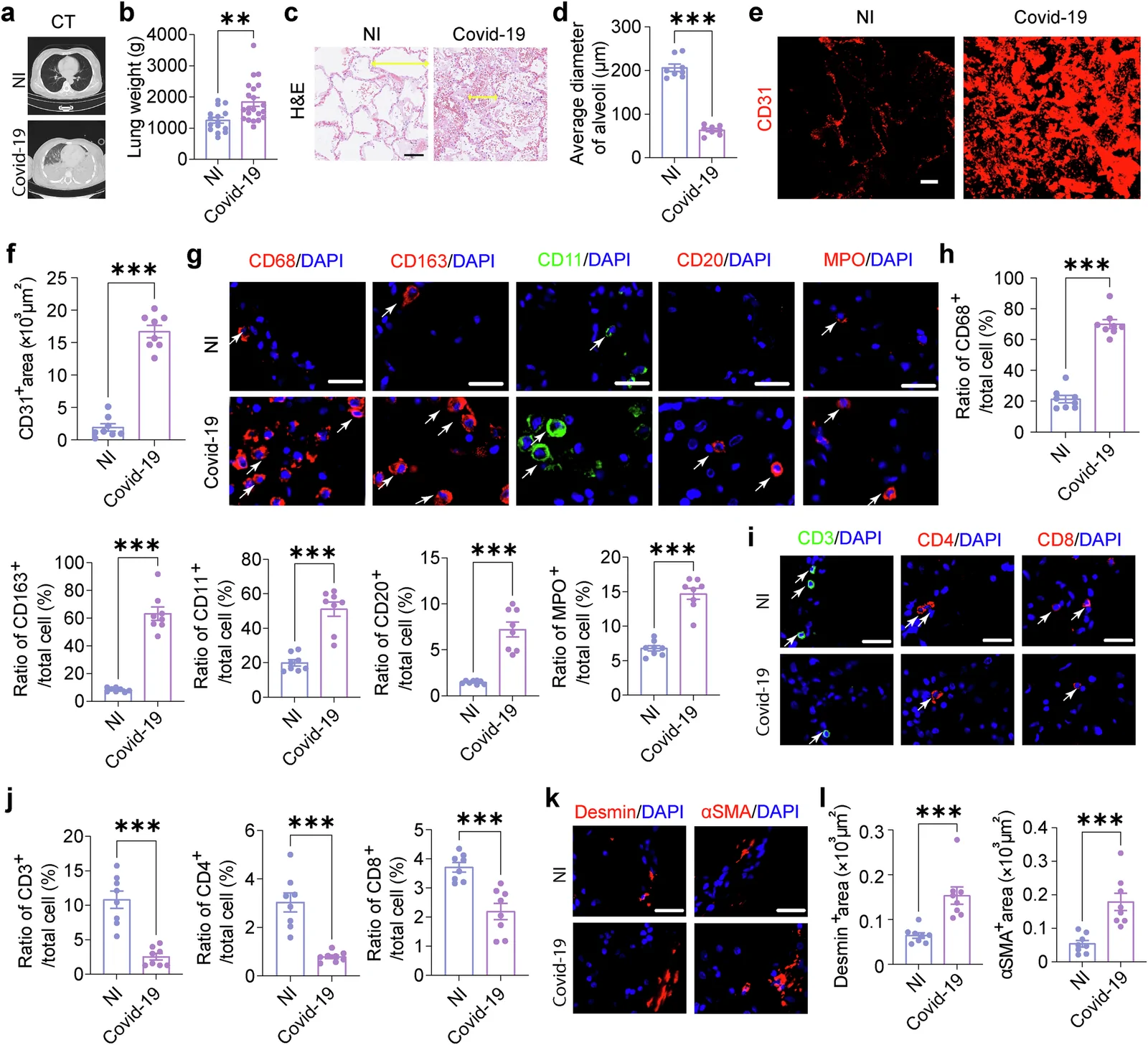
Lung pathology of COVID-19 patients and noninfected individuals. **a** Representative chest CT images from noninfected individuals and COVID-19 patients. **b** Lung weight measurements comparing NI and COVID-19 cases (*n* = 15–21 per group). **c** H&E-stained lung sections of NI and COVID-19 patients. **d** Quantification of alveolar diameter in NI and COVID-19 lung tissues (n = 8 fields per group). **e** CD31 immunohistochemical staining in lung tissues of NI and COVID-19 patients. **f** Quantification of the CD31-positive area of NI and COVID-19 lung tissues (n = 8 fields per group). **g** Immunohistochemical staining of macrophage markers (CD68, CD163), a dendritic cell marker (CD11), a B-cell marker (CD20), and a neutrophil marker (MPO) in lung sections in NI and COVID-19 lung sections. **h** Comparative quantification of CD68^+^, CD163^+^, CD11^+^, CD20^+^, and MPO^+^ cells between NI and COVID-19 lung tissues (*n* = 8 fields per group). **i** T-cell marker staining (CD3, CD4, CD8) in NI and COVID-19 lung sections. **j** Quantification of CD3^+^, CD4^+^, and CD8^+^ cells in NI and COVID-19 lung tissues (*n* = 8 fields per group). **k** Immunofluorescence staining of stromal fibroblast and myofibroblast markers (Desmin, αSMA) in NI and COVID-19 lung tissues. **l** Quantification of desmin- and αSMA-positive areas in NI and COVID-19 lung tissues (*n* = 8 fields per group). Data are presented as the mean ± s.e.m. *p < 0.05, **p < 0.01, ***p < 0.001; two-sided unpaired t test. Scale bars = 50 μm

To relate our preclinical findings to human relevance, we collected lung tissues from healthy lung tissues and SARS-CoV-2-infected human individuals by autopsy analysis. A total of 15 adult individuals (6 males and 9 females) died of acute myocardial infarction, acute circulatory failure, or septic shock. None of these patients had COVID-19 at the time of autopsy analysis. Upon autopsy examination, their lung tissues were collected as the non-COVID-19 group. The demographic information of these non-COVID-19 individuals is shown in Suppl. table 1. A cohort of 21 adult individuals (15 males and 6 females) who died of severe COVID-19 during 2020 and 2021 were also recruited for our study. CT examination showed a typical lung image of SARS-CoV-2-infected lung tissue that exhibited high-density imaging of consolidation with bilateral, multilobar ground-glass opacification with a peripheral or posterior distribution (Fig. 5a). It should be emphasized that these patients suffered severe COVID-19 pulmonary disease and eventually died of acute respiratory distress syndrome (ARDS). Their lung weights were significantly increased relative to those of the non-COVID-19 group (Fig. 5b). Histological examination further validated the infiltrated cellular and noncellular components of the entire pulmonary tissues (Fig. 5c, d). In particular, the alveolar space was filled with cells and extravasated plasma.

Importantly, SARS-CoV-2-infected human pulmonary tissues contained exceptionally high numbers of microvessels that appeared to be disorganized, tortuous, interconnected plexus, and nonuniform in size (Fig. 5e, f). These distinct vascular changes were reminiscent of those observed in SARS-CoV-2-infected mouse lung tissues (Fig. 1). Additionally, such disorganized vascular networks were often observed in malignant tumor tissues, particularly in high VEGF-expressing tumor tissues (Fig. 3h, j). According to our experience, VEGF is highly responsible for inducing such disorganized blood vessel networks. For example, implantation of a VEGF-embedded slow-release polymer in the mouse cornea also instigates the formation of such disorganized vascular plexuses.^46^ Furthermore, one of our previous clinical studies showed that anti-VEGF treatment of human patients with severe COVID-19 markedly improves pulmonary functions and clinical outcomes.^31^

In addition to vascular changes, SARS-CoV-2 infection also augmented severe pulmonary inflammation by increasing infiltration of the total population of myeloid cells, monocytes/macrophages, and neutrophils (Fig. 5g, h). In contrast, the CD3^+^ total T-cell population, the CD8^+^ cytolytic T-cell subpopulation and the CD4^+^ helper T-cell population were all significantly reduced (Fig. 5I, j), indicating the existence of severe lymphopenia in these patients with severe COVID-19. The inflammatory phenotype of these COVID-19 patients is in general agreement with what has been reported by others.^47^ Furthermore, the COVID-19 lung tissues also contained increased fibrotic components (Fig. 5k, l).

### Severe tissue hypoxia and elevated VEGF expression in SARS-CoV-2-infected lungs

**Fig. 6:**
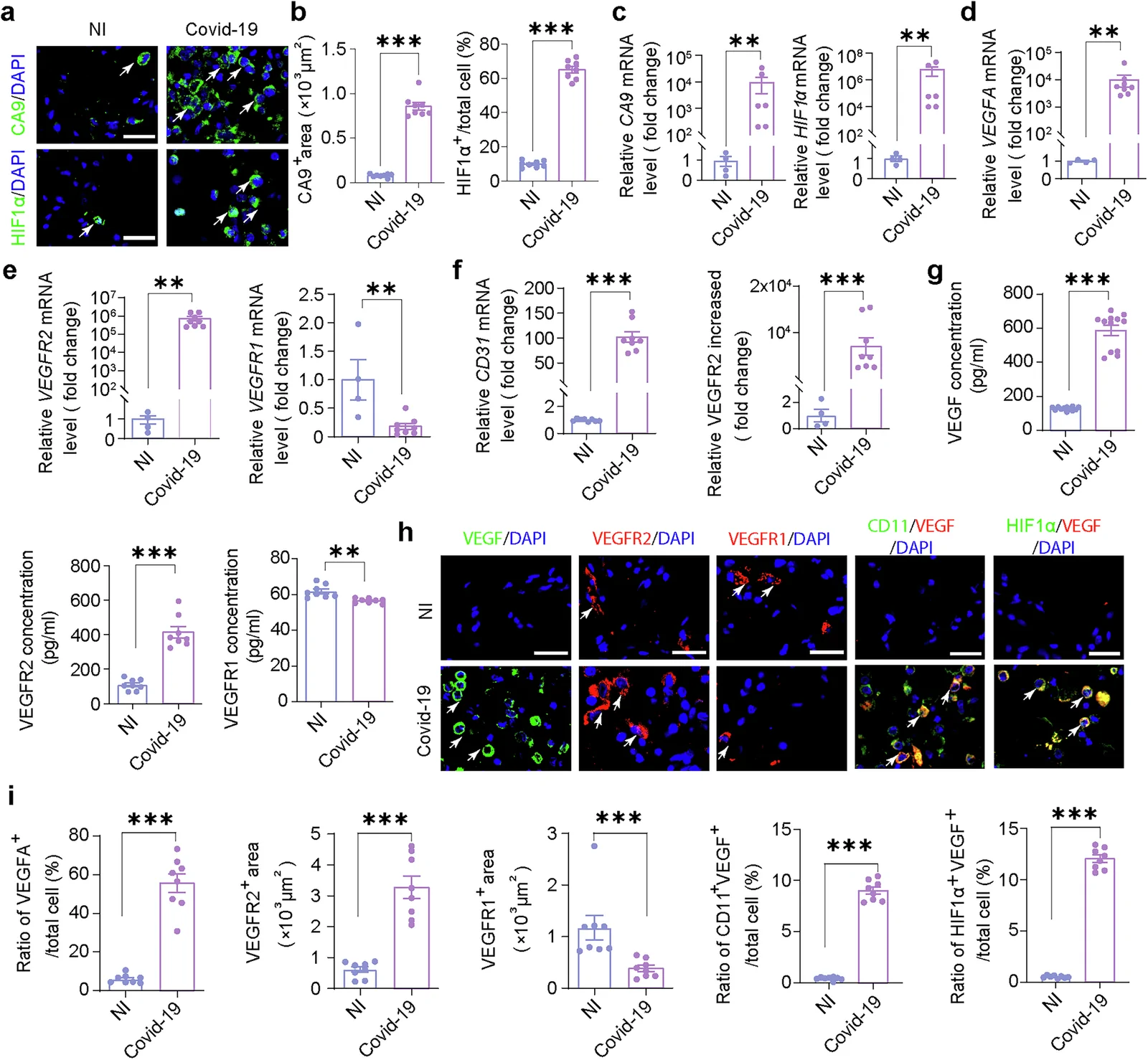
Hypoxic response and VEGF signaling in human lung tissues. **a** Representative immunofluorescence staining of the hypoxia markers HIF1α (green) and CA9 (green) in lung sections from NI and COVID-19 patients. Nuclei are counterstained with DAPI (blue). **b** Quantification of HIF1α-positive cells and CA9-positive area in NI and COVID-19 patient lung tissues (*n* = 8 fields per group). **c** Relative mRNA expression levels of HIF1α and CA9 in NI and COVID-19 patient lung tissues (*n* = 4–6 samples per group). **d** Relative mRNA expression levels of VEGFA in NI and COVID-19 patient lung tissues (*n* = 4–8 samples per group). **e** Relative mRNA expression levels of VEGFR2 and VEGFR1 in NI and COVID-19 patient lung tissues (*n* = 4–8 samples per group). **f** Relative mRNA expression levels of CD31 and the relative fold increase in VEGFR2 expression in NI and COVID-19 patient lung tissues (*n* = 4–8 samples per group). **g** Protein concentrations of VEGFA, VEGFR2, and VEGFR1 measured by ELISA in lung tissue homogenates of NI and COVID-19 cases (*n* = 8–12 samples per group). **h** Representative immunohistochemical staining of VEGFA, VEGFR2, VEGFR1, CD11^+^ VEGF^+^, and HIF1α^+^ VEGF^+^ in lung sections of NI and COVID-19 patients. **i** Quantitative analysis of VEGFA-positive cells, CD11 and VEGF double-positive cells, HIF1α and VEGF double-positive cells, and VEGFR2/VEGFR1-positive areas between NI and COVID-19 lung tissues (*n* = 8 fields per group). Data are presented as the mean ± s.e.m. *p < 0.05, **p < 0.01, ***p < 0.001; two-sided unpaired t test. Scale bars = 50 μm

To further correlate our preclinical findings from mouse models to human relevance, we quantitatively measured tissue hypoxia and VEGF expression in the COVID-19 lung tissues of human patients. Similar to SARS-CoV-2-infected mouse lung tissues, human COVID-19 lung tissues also suffered from severe hypoxia by markedly increasing CA9 and HIF1α expression (Fig. 6a). Approximately a 9-fold increase in CA9 and a 4-fold increase in HIF1α were discovered in COVID-19 lung tissues relative to their controls (Fig. 6b). Concordantly, quantitative PCR (qPCR) analysis corroborated the marked increases in CA9 and HIF1α mRNA levels in COVID-19 lung tissues (Fig. 6c). These results show the existence of severe hypoxia in SARS-CoV-2-infected human lung tissues.

The presence of severe pulmonary hypoxia prompted us to investigate hypoxia-induced downstream signaling molecules such as VEGF. Reconciling with severe hypoxia and high levels of HIF1α, the VEGF mRNA level was also markedly increased (Fig. 6d). Interestingly, VEGFR2, the functional receptor responsible for most VEGF-induced biological activities, was also increased (Fig. 6e). In contrast, VEGFR1, which has been considered a decoyed negative receptor for VEGF signaling, was markedly decreased (Fig. 6e). In support of this line of quantitative results, quantification of the endothelial cell marker CD31 demonstrated an approximate 100-fold increase in SARS-CoV-2-infected human lung tissues relative to non-COVID-19 lung tissues (Fig. 6f). In concordance with the mRNA expression levels, the pulmonary protein levels of VEGF, VEGFR2, and VEGFR1 were accordingly up- and downregulated (Fig. 6g). Immunohistochemical staining of lung tissues further strengthened our conclusions of elevated VEGF and VEGFR2 and decreased VEGFR1 expression in SARS-CoV-2-infected human lung tissues relative to controls (Fig. 6h, i). Taken together, these human results support the facts of COVID-19-induced severe pulmonary hypoxia, exceptionally high expression of VEGF and VEGFR2, and angiogenesis in SARS-CoV-2-infected human lung tissues.

### Anti-VEGF therapy blocks COVID-19-triggered tumor outgrowth

**Fig. 7:**
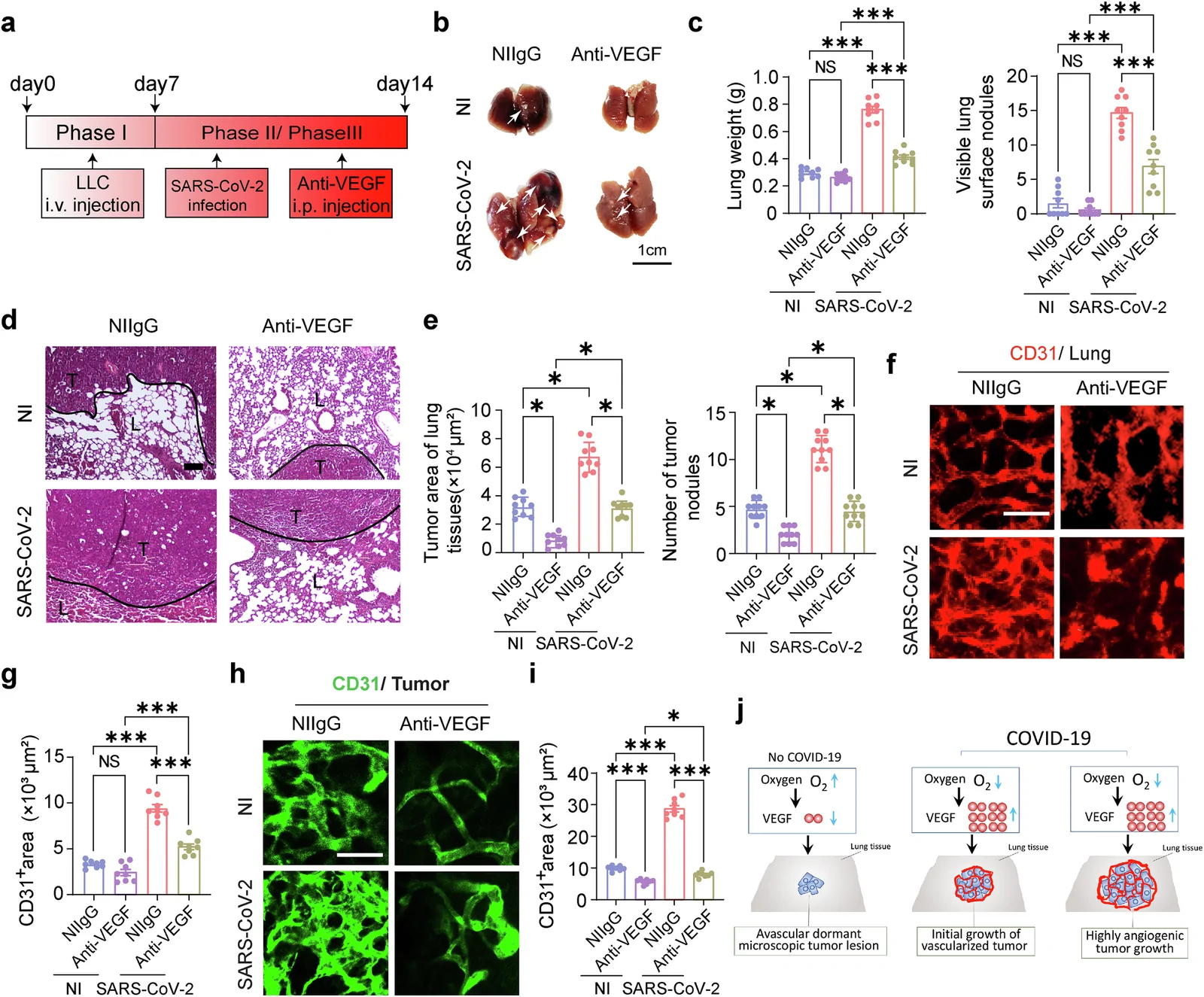
Anti-VEGF therapy blocks COVID-19-triggered dormant tumor growth. **a** Experimental timeline showing SARS-CoV-2 (SC) infection, administration of anti-VEGF antibody or NIIgG, and sample collection. **b** Representative gross images of lungs and tumors from NIIgG- and anti-VEGF-treated NI- or SC-infected mice. **c** Quantification of lung weight and visible tumor nodules in NIIgG- and anti-VEGF-treated NI- or SC-infected animals (*n* = 9 per group). **d** Representative H&E staining of lung sections showing tumor regions (T) and adjacent lung tissues (L) from NIIgG- and anti-VEGF-treated NI- or SC-infected mice. **e** Quantification of tumor area and size (*n* = 9–10 per group). **f** Immunofluorescence staining of CD31 (red) in peritumoral lung tissues showing SC infection–induced hypervascularization and its reduction by anti-VEGF therapy. **g** Quantification of CD31⁺ microvessel density in peritumoral lung tissues (*n* = 8 fields per group). **h** CD31 staining (green) of tumor vasculature showing dense, tortuous, and disorganized microvessels in SC-infected mice and vascular normalization upon anti-VEGF treatment. **i** Quantification of CD31⁺ microvessel density in tumor regions (*n* = 8 fields per group). **j** Hypothesis of SARS-CoV-2–induced pulmonary hypoxia promoting VEGF upregulation and tumor angiogenesis. Data are presented as the mean ± s.e.m. *p < 0.05, **p < 0.01, ***p < 0.001; one-way ANOVA with Tukey’s post hoc test. NS not significant. Scale bars = 50 μm

To determine VEGF-dependent tumor outgrowth by SARS-CoV-2 infection, we used a pharmacological approach of anti-VEGF therapy. VEGF blockade was used in our in vivo experimental settings. This experiment was divided into three phases (Fig. 7a): (1) Phase I: 1 × 10^6^ LLC tumor cells were intravenously injected into each of the male heterozygous K18-hACE C57BL/6 J mice; (2) Phase II. After 7 days of tumor implantation, SARS-CoV-2 was used to infect each of the tumor-bearing mice. (3) Phase III. Shortly after SARS-CoV-2 infection, VEGF blockade was administered to each tumor-bearing mouse. Non-SARS-CoV-2-infected animals and nonimmune IgG were used as controls. Finally, after 2 weeks of tumor cell implantation and 1 week of SARS-CoV-2 infection, tumor-bearing mice were sacrificed, and tumor tissues and various organs were dissected for further analysis (Fig. 7a).

According to this experimental design, treatment of SARS-CoV-2-infected tumor-implanted mice with VEGF blockade markedly decreased lung tumor formation and tumor growth (Fig. 7b, c). Gross examination of lung tissues showed that SARS-CoV-2 infection overtly augmented tumor formation and growth relative to the noninfected controls (Fig. 7b, c). The tumor-promoting effect of COVID-19 was virtually blocked by VEGF blockade (Fig. 7b, c). H&E staining of lung tissues reconciled the existence of high numbers and large tumor lesions in the SARS-CoV-2-infected group (Fig. 7d, e). Again, anti-VEGF treatment reduced both tumor numbers and mass in this experimental setting (Fig. 7d, e). Many of these anti-VEGF-treated tumors in the SARS-CoV-2-infected group remained in their tiny microscopic lesions, a typical phenotype seen in tumor dormancy. These results show a VEGF-dependent mechanism of SARS-CoV-2-initiated tumor formation and growth.

### Anti-VEGF treatment blocks COVID-19-induced pulmonary and tumor angiogenesis

To investigate the vascular responses of pulmonary tissues to anti-VEGF treatment, lung tissues from healthy mice and SARS-CoV-2-infected animals were analyzed for vascular changes. While anti-VEGF treatment had no impact on pulmonary microvessels in non-COVID-19 animals, VEGF blockade markedly reduced the vascular density of SARS-CoV-2-infected lung tissues (Fig. 7f, g). In addition to decreases in vascular density, anti-VEGF treatment also remodeled pulmonary vasculatures toward vascular normalization, a phenotypic change often seen in anti-VEGF-treated tumors.^48^

Similar to lung tissues, tumor vessels in non-COVID-19 and COVID-19 animals were highly sensitive to anti-VEGF treatment (Fig. 7h, i). However, tumors grown in SARS-CoV-2-infected animals contained an exceptionally high density of microvessels, which were disorganized and tortuous (Fig. 7h). These disorganized vascular plexuses were typical VEGF^high^ tumor vasculatures that lacked separation of each individual microvessel (Fig. 7h). Anti-VEGF treatment reduced the vessel density and improved vascular structures (Fig. 7h, i). These data show that SARS-CoV-2 infection switches to pulmonary hypoxia that augments VEGF expression and tumor angiogenesis (Fig. 7j).

**Fig. 8:**
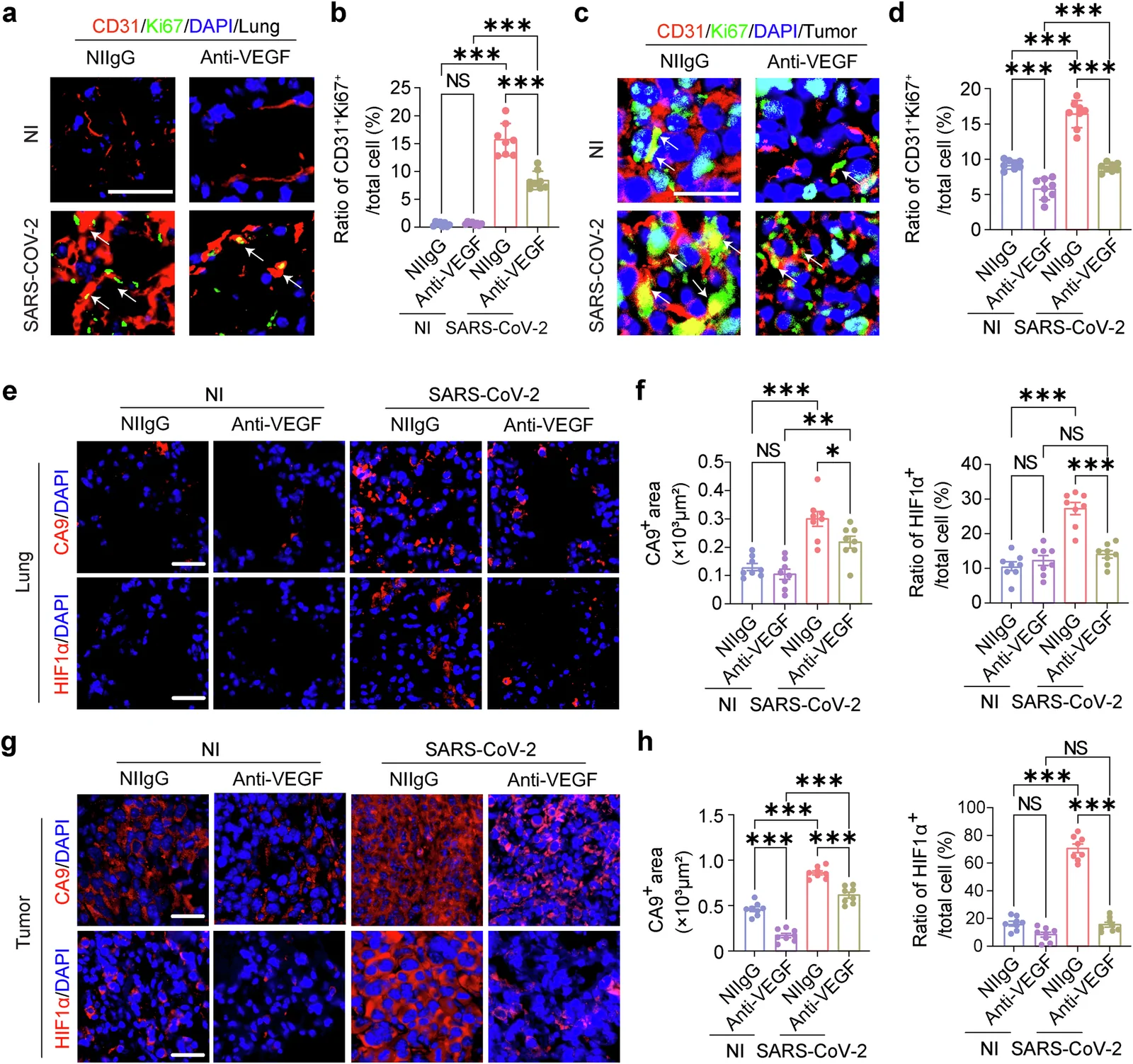
Anti-VEGF therapy inhibits COVID-19-induced tumor angiogenesis. **a** Representative immunofluorescence images showing costaining of CD31 (red) and Ki67 (green) in peritumoral lung tissues from NIIgG- and anti-VEGF-treated NI- or SC-infected mice. **b** Quantification of Ki67⁺/CD31⁺ proliferating endothelial cells in peritumoral lung tissues (*n* = 8 fields per group). **c** Costaining of CD31 (red) and Ki67 (green) in tumor tissues from NIIgG- and anti-VEGF-treated NI- or SC-infected mice. **d** Quantification of Ki67⁺/CD31⁺ double-positive endothelial cells in tumor regions (*n* = 8 fields per group). **e** Immunofluorescence staining of CA9 and HIF1α (red) in peritumoral lung tissues showing COVID-19-induced hypoxia and its attenuation by anti-VEGF treatment. **f** Quantification of CA9⁺ and HIF1α⁺ signals in peritumoral lung tissues (*n* = 8 fields per group). **g** Immunofluorescence staining of CA9 and HIF1α (red) in tumor tissues from NIIgG- and anti-VEGF-treated NI- or SC-infected mice. **h** Quantification of CA9⁺ and HIF1α⁺ signals in tumor regions (*n* = 8 fields per group). Data are presented as the mean ± s.e.m. *p < 0.05, **p < 0.01, ***p < 0.001; one-way ANOVA with Tukey’s post hoc test. NS not significant. Scale bars = 50 μm

Consistent with increased vascular density, microvascular endothelial cells in SARS-CoV-2-infected lung tissues were highly proliferative and exhibited CD31^+^ and Ki67^+^ double positivity (Fig. 8a). Again, the proliferating endothelial population was markedly reduced by VEGF blockade (Fig. 8a, b). These findings show that COVID-19-triggered pulmonary angiogenesis is dependent on VEGF. Likewise, the numbers of proliferating endothelial cells were markedly reduced in tumors grown in both non-COVID-19 and COVID-19 animals (Fig. 8c, d). In parallel, both pulmonary and tumor tissues from anti-VEGF-treated animals exhibited reduced Ki67^+^ proliferation and TUNEL^+^ apoptosis, indicating attenuation of COVID-19-induced cellular turnover (Suppl. Fig. 10b and 11).

Along vascular changes, anti-VEGF treatment markedly improved COVID-19-induced lung hypoxia and tumor hypoxia (Fig. 8e–h). Noticeably, the HIF1α protein level in the anti-VEGF-treated tumors grown in COVID-19 animals returned to the basal level, which was indistinguishable from that of the control tumors (Fig. 8h). Consistently, CD3^+^, CD4^+^, and CD8^+^ T-cell populations in the lungs were restored after anti-VEGF therapy, reflecting partial reversal of COVID-19-associated lymphopenia (Suppl. Fig. 12). In tumors, VEGF blockade also enhanced T-cell infiltration and normalized CD3^+^, CD4^+^, and CD8^+^ subsets (Suppl. Fig. 13). Furthermore, both F4/80^+^ macrophages and their M1-like (CD80^+^) and M2-like (CD206^+^) subsets were normalized in the lungs and tumors after anti-VEGF treatment, suggesting suppression of inflammatory macrophage polarization (Suppl. Figs. 14 and 15). In addition, the abundance of FAP^+^, desmin^+^, and αSMA^+^ fibroblasts and myofibroblasts was markedly decreased in both pulmonary and tumor tissues, indicating attenuation of fibrosis and stromal remodeling under VEGF blockade (Suppl. Figs. 16 and 17). These data further support our conclusion of VEGF-dependent tumor angiogenesis and outgrowth in the SARS-CoV-2-infected animal model.

## Discussion

The concept of angiogenesis-dependent tumor growth remains an undisputed dogma and has paved the therapeutic avenue for antiangiogenic cancer therapy.^49^ Avascular tumors may remain tiny in size without further growth, often existing as microscopic clusters of cancer cells.^13,15,16,50^ Microscopically dormant tumor nodules are often located in the vicinity of preexisting blood vessels where they obtain nutrients and signaling molecules for the maintenance of survival.^13,15,16^ Malignant cells in dormant tumors may remain quiescent by entering G0/G1 cell cycle arrest.^3,4^ Alternatively, cancer cell populations in dormant tumors may relentlessly renew themselves by a balance between proliferation and cell death.^15^ Renewal of cancer populations would provide an opportunity to gain new genetic mutations and epigenetic changes, which eventually lead to switching to an angiogenic phenotype that supports tumor outgrowth.^51^ In support of this view, mutations of oncogenic Ras and tumor suppressor p53 have been linked to tumor angiogenesis by producing various angiogenic factors.^43,52,53^

**Fig. 9:**
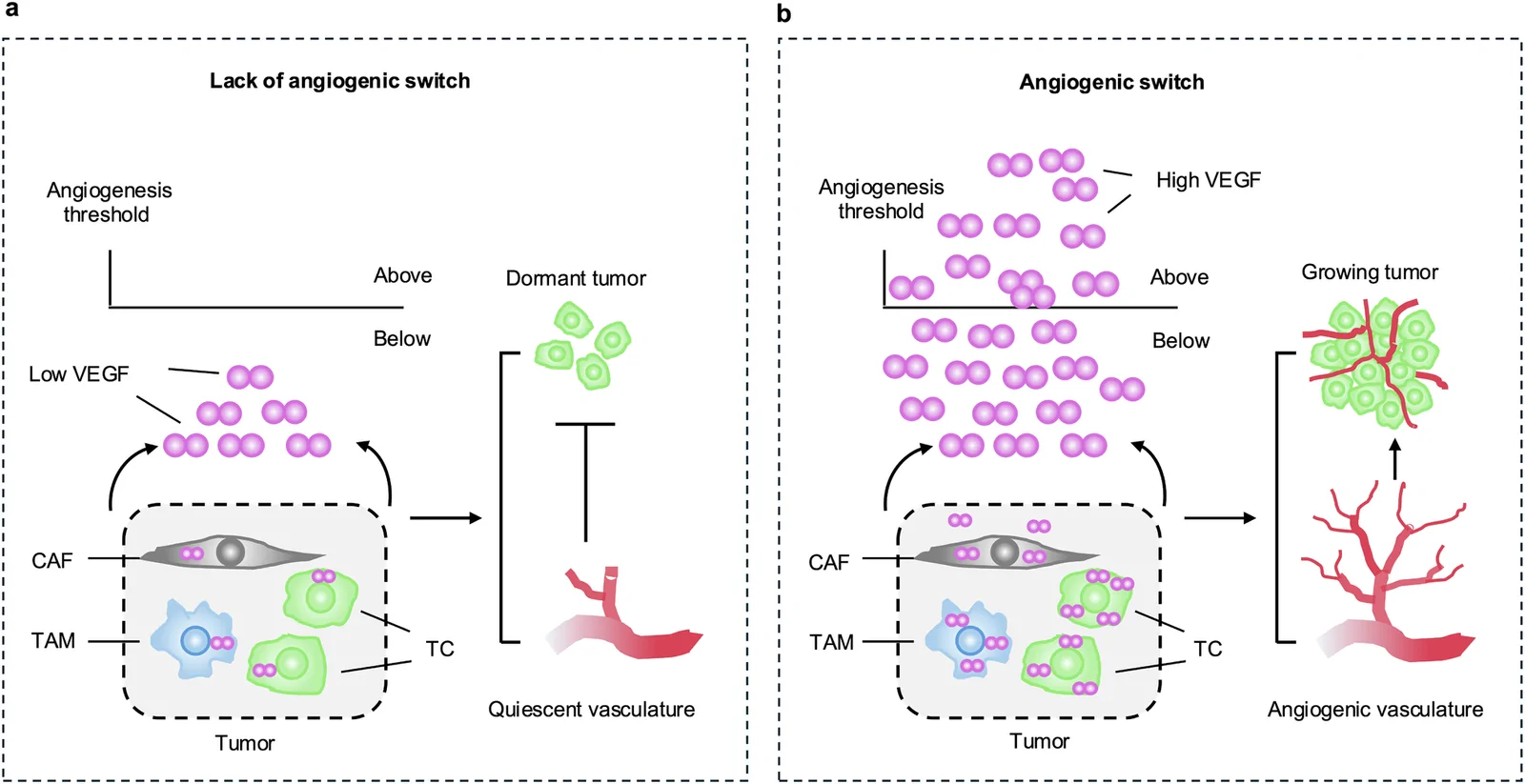
Schematic diagram of VEGF-dependent tumor initiation and a hypothetical threshold of an angiogenic switch. **a** In dormant tumors, the levels of proangiogenic factors derived from tumor cells and other stromal cells are very low, which incapacitate switching to an angiogenic phenotype. In this avascular microenvironment, tumor cells remain in their dormant stage and lack growth into clinically detectable tumor masses. The low level of proangiogenic factors is defined below the angiogenic threshold. Angiogenic factors in the TME can be produced by malignant cells and other stromal cells, including inflammatory cells and cancer-associated fibroblasts. **b** If the levels of proangiogenic factors in the TME reach beyond the angiogenic threshold, tumor angiogenesis actively occurs and initiates dormant tumor growth. VEGF molecules are used as an example of proangiogenic factors. It is likely that other non-VEGF proangiogenic factors also actively participate in the switch to an angiogenic phenotype. VEGF vascular endothelial growth factor, CAF cancer-associated fibroblast, TAM tumor-associated macrophage, TC tumor cell

We hypothesize that the amount of angiogenic factors in the TME should reach beyond a hypothetical threshold level to induce tumor angiogenesis (Fig. 9). These tumor angiogenic factors can be produced by local cancer cells and stromal cells, including cancer-associated fibroblasts and tumor-associated macrophages, and distant tissue-derived circulating factors.^54^ When low levels of angiogenic factors are below the threshold, blood vessels remain quiescent without growth (Fig. 9). In this case, tumors continue to remain avascular without further growth.^54^ However, if angiogenic stimuli such as VEGF in the TME endure beyond the threshold level, neovascularization begins by sprouting from preexisting vessels. To test this hypothesis, we genetically propagated cancer cells by producing a high level of VEGF. In a mouse tumor model, we have provided compelling evidence to show that high levels of VEGF promote tumor outgrowth. We emphasize that our mouse LLC tumor model is not a clinically relevant tumor dormancy model. However, this preclinical model provides important clues to study tumor outgrowth and dormancy in relation to alterations of the TME. One of the puzzling scenarios in experimental tumor models is the implantation of a relatively large number of cancer cells in animals, usually millions of cells, to ensure the formation of a tumor. Why would such a high number of tumor cells be needed for tumor formation? Several hypotheses, including cancer stem cells, may explain this interesting enigma. However, the commonly used cancer cell lines are less likely to entail cancer stem cell issues. We reasonably propose a hypothesis that a high number of cancer cells are required to produce sufficient angiogenic molecules beyond the angiogenic threshold level to switch on angiogenesis and support tumor outgrowth (Fig. 9). In a tumor cell-titration experiment, we demonstrated that only 1 × 10^4^ VEGF^high^-expressing tumor cells are needed to initiate tumor outgrowth. In contrast, very high numbers of low VEGF-expressing tumor cells are needed to instigate tumor formation and outgrowth. These findings support the concept of the prerequisite role of an angiogenic switch in supporting tumor outgrowth.

We also admit some limitations of generalizing our findings for all tumors because multiple variations and unpredictable factors exist in various tumors, including the following: (1) Variations in genetic and epigenetic alterations exist in various tumors, which could alter the threshold levels of proangiogenic factors; (2) Variations in the stromal cellular compositions in various tumors could also alter the angiogenic threshold level; and (3) Variation in the production of different angiogenic factors could also be a key determinant for an angiogenic switch. It is known that many angiogenic factors can produce synergistic effects for neovascularization. Thus, the composition of various proangiogenic factors is crucial for an angiogenic switch. (4) Vascular heterogeneity. Threshold levels of proangiogenic factors for neovascularization could vary markedly in various tissues owing to their sensitivity to various proangiogenic factors. Endothelial cells in various tissues are highly heterogeneous. Thus, a threshold level of proangiogenic factors for neovascularization should be subjected to changes in the context of tumor types, TME, and tissue locations where tumors rise. Although the LLC tumor model was used to investigate tumor outgrowth, there are several limitations of this preclinical model to relate to clinical tumor dormancy, including the following: (1) The experimental duration is rather short to study tumor dormancy. In clinical settings of tumor dormancy, individuals with dormant tumors usually experience nonclinically manifested cancer disease for months, years, and even decades. In our mouse COVID-19 model, the entire experimental duration lasts only a couple of weeks. (2) The LLC tumor model is a relatively aggressive tumor model for studying tumor dormancy. In this respect, the LLC tumor model is still far from true clinical tumor dormancy. (3) The tail-vein implantation model is less relevant to clinically spontaneous tumor dormancy, which occurs spontaneously in humans. (4) The mouse LLC model typically allows us to investigate tumor outgrowth rather than awakening dormant tumors. (5) As tumor dormancy entails complex mechanisms of immunity, angiogenesis, and tumor cell mutations, our mouse model is a rather simplified system to investigate tumor dormancy. (6) Owing to strict P3 laboratory regulations, many important experiments, including single-cell RNA sequencing and fluorescence-activated cell sorting (FACS) analysis, have not been performed in our study. Therefore, our conclusions are limited and mainly based on the available methodologies. Nevertheless, despite these limitations, our findings using the experimental LLC model in COVID-19 mice provide important clues to investigate tumor outgrowth in relation to an angiogenic switch. High mRNA levels of VEGF and VEGFR2 were detected in the lung tissues of human patients with severe COVID-19. However, owing to experimental limitations in handling human SARS-CoV-2-infected tissue samples, we are unable to provide quantification data of VEGF and VEGFR2 protein levels. According to our experience with mouse samples, a significant difference exists between mRNA levels and protein levels of VEGF, and the protein levels are generally much lower than mRNA levels (Fig. 1). In addition to hypoxia-induced high VEGF production, we believe that pulmonary inflammation also significantly contributes to high VEGF production. In support of this view, VEGF-positive signals were also localized to inflammatory cells, including macrophages (Fig. 6h). When this ligand‒receptor signaling is dictated by biological functions, an explosive and overwhelming angiogenic response exists in SARS-CoV-2-infected lung tissues. Indeed, our data show the existence of an exceptionally high density of microvessels in COVID-19 lung tissues. Imaging a dormant tumor in such a hypervascular bed of lung tissues, it would not be surprising that a dormant avascular tumor begins its growth in the COVID-19-propagated vascular bed. Therefore, treatment of severe COVID-19 not only alleviates COVID-19-associated pulmonary pathologies and clinical symptoms but also effectively prevents tumor outgrowth during the long COVID-19 period. Thus, anti-VEGF therapy would have profound impacts on the functional improvement of lung tissues and lung cancer prevention during and after the COVID-19 periods. In a mouse COVID-19 model, we detected exceptionally high VEGF levels in infected lung tissues and plasma.^39^ Since VEGF molecules are synthesized in various isoforms owing to alternative splicing of mRNA, the high-molecular forms of VEGFs are sequestered by heparan sulfate proteoglycans in the local extracellular matrix. Thus, only smaller and non-heparin-binding VEGF isoforms enter the circulation.^55^ In this regard, the VEGF concentration in pulmonary tissues should be higher than that in plasma. Indeed, an exceptionally high level of VEGF was detected in SARS-CoV-2-infected lung tissues. Both local and circulating VEGFs contribute to an angiogenic switch in pulmonary tissue, which initiates and supports the growth of dormant tumors.^18^ The upstream driver of VEGF-induced angiogenesis is tissue hypoxia, a pathological alteration commonly seen in severe COVID-19.^56^

If the hypoxia/inflammation-VEGF-angiogenesis axis is the primary trigger for initiating tumor outgrowth, our findings could be further expanded to other non-COVID-19-pathological settings, including infection, inflammation, exposure to hypoxia, smoke, inhalation of toxic chemicals, pulmonary tissue damage, and obstruction of the upper respiratory tract. In fact, these factors have been known to increase the risk of lung cancer development.^57,58^ Perhaps the hypoxia/inflammation-VEGF-angiogenesis-initiated tumor outgrowth provides new mechanistic insights into high lung cancer incidences under these conditions. Another important cellular source of high VEGF production in SARS-CoV-2-infected pulmonary tissue is inflammatory cells. It is known that myeloid-derived inflammatory cells, including monocytes/macrophages and neutrophils, produce high levels of VEGF during SARS-CoV-2 infection.^56^ It is plausible that VEGF may have direct effects on tumor cell proliferation, which subsequently contributes to an angiogenic switch. This possible mechanism may restrain certain types of cancer because not all cancer cells are responsive to VEGF stimulation. Previous studies have shown that SARS-CoV-2 infection induces cellular senescence and sustained senescence-associated inflammatory responses in the lung.^59^ Senescent cells may secrete a variety of SASP factors, including VEGF, IL-6, and IL-8, thereby contributing to angiogenesis, inflammation, and tissue remodeling. Therefore, VEGF upregulation in the SARS-CoV-2-infected lung may not be exclusively driven by highly proliferative tumor cells but may also involve senescence-associated epithelial or stromal cells. These findings suggest that proliferative and senescent phenotypes may coexist in the postinfectious lung microenvironment and jointly contribute to metastatic progression and VEGF induction. Future studies incorporating additional senescence markers, such as SA-β-gal, will help to further clarify the contribution of senescence to VEGF upregulation after SARS-CoV-2 infection. In addition to angiogenesis, immune suppression offers another plausible mechanism for initiating tumor outgrowth. VEGF plays multifarious functions to suppress the immune response,^60,61^ including (1) inhibition of CD8^+^ T-cell proliferation and migration; (2) promotion of CD8^+^ T-cell exhaustion; (3) inhibition of CD8^+^ T recruitment in tumors; (4) stimulation of Treg proliferation and recruitment; (5) recruitment of myeloid-derived suppressive cells; and (6) inhibition of dendritic cell maturation. On the basis of the VEGF-mediated complex mechanisms of immune suppression, it is perhaps unsurprising to speculate that immunosuppression significantly participates in the initiation of tumor outgrowth. Collectively, an angiogenic switch and immunosuppression may synergistically contribute to accelerated tumor growth in our experimental models. Hypoxia has been linked to cell cycle arrest and the development of tumor dormancy in other experimental settings.^62^ Perhaps the level of hypoxia is the key determinant for switching to an angiogenic phenotype in tumors. In our experimental SARS-CoV-2 mouse models, the hypoxic level was high. In addition to hypoxia, SARS-CoV-2 infection augments severe lung inflammation that also contributes to high VEGF production and angiogenic switching. In fact, VEGF synthesis has been largely localized to inflammatory cells, including macrophages (Fig. 6). It is plausible that in other hypoxia models and experimental settings, inflammation is not a confounding factor in studying tumor dormancy. Our findings of the hypoxia- and inflammation-VEGF axis in promoting tumor outgrowth may not be limited to SARS-CoV-2-infected lung tissues. As the key mechanism of tumor outgrowth in our experimental model is determined by hypoxia/inflammation-VEGF-angiogenesis signaling, any other pathogens, such as severe influenza, bacterial infection, ARDS of various etiologies, and other inflammatory lung injuries, are also likely to activate this signaling pathway to trigger tumor outgrowth. Thus, the concept of triggering tumor outgrowth based on our findings may well be generalized to other etiologies in relation to pulmonary hypoxia and inflammation. These interesting possibilities warrant future investigation.

On the basis of these discoveries, we reasonably propose an interventional paradigm for preventing tumor outgrowth. Perhaps a low-dose anti-VEGF drug lacking overt adverse effects but maintaining its level below the threshold can be used for prevention or treatment of dormant tumor outgrowth by inactivating an angiogenic switch. In particular, this approach is potentially effective for preventing postoperative recurrence and metastatic growth.

## Materials and methods

### Ethical permission for animal and human studies

All animal experiments were conducted in accordance with the approved ethical permit (permit number 10712-2020). The animals were housed at the Astrid Fagraeus laboratory, and the studies were performed in a biosafety level 3 facility at the Karolinska Institutet, following the regulations set by the Swedish Board of Agriculture. The animals were kept on a 12 h dark cycle. They had unrestricted access to a diet (CRM; 801722; Special Diets Services). The experimental procedures were approved by the local ethics committee, Stockholms Norra Djurförsöksetiska Nämnd, and complied with the ARRIVE guidelines (Animal Research: Reporting of In Vivo Experiments). All animals were randomly divided into groups to receive various treatments. For the human studies, all autopsies were performed at the risk-autopsy facility of the Department of Clinical Pathology/Cytology, Karolinska University Hospital, Huddinge, Stockholm, Sweden. Subjects were referred to the pathology department for clinical autopsies to determine the exact cause of death. The study was approved by the Swedish Ethical Review Authority under approval numbers DNR 2020-02446 and 2020-04339.

### Animal COVID-19 models and drug treatment

SARS-CoV-2 infection in mouse models and drug treatments were performed according to our previously published methods.^39^ Briefly, heterozygous K18-hACE C57BL/6 J mice (strain: 2B6. Cg-Tg(K18-ACE2) 2Prlmn/J) were obtained from Jackson´s Laboratory (Strain #034860). The animals were housed in groups of fewer than five per cage and were fed a standard chow diet. At 12 weeks of age, the mice were intranasally administered ‘wild-type’ (the original strain) SARS-CoV-2 virus at a dose of 1 × 10^2^ p.f.u. (plaque-forming unit) per mouse. The SARS-CoV-2 viruses were isolated and expanded from clinical samples of wild-type SARS-CoV-2 provided by Prof. Gerald McInerney’s group at the Karolinska Institute, following previously published protocols.^63^ A rabbit anti-mouse VEGF neutralizing antibody (BD0801, Simcere Pharmaceutical Company, Nanjing, China) was intraperitoneally administered to each mouse at a dose of 7.5 mg kg^−1^ every other day, starting on day 0 of SARS-CoV-2 infection. Animals treated with nonimmune IgG (NIIgG) antibody served as controls and received the same treatment regimen. All animal experiments were terminated using a lethal dose of isoflurane.

### Mouse tumor models

Mouse tumor models were established according to our previously published protocols.^64^ Briefly, LLC-vec (vector-transfected LLC cells) and LLC-VEGF (LLC cells stably expressing human hVEGF)^65^ were suspended at the indicated cell concentrations in 100 μl sterile PBS and implanted into 8-week-old C57BL/6NRj mice either subcutaneously or intravenously via the tail vein according to the experimental design. Tumor dimensions were measured using calipers every other day, and tumor volumes were calculated as (length × width × width × 0.52).^66^ For VEGF blockade studies, a rabbit anti-mouse VEGF neutralizing antibody (BD0801, Simcere Pharmaceutical Company, Nanjing, China)^43–45^ or nonimmune IgG control was delivered intraperitoneally at 7.5 mg kg⁻¹ every other day, initiated 7 days after tumor inoculation and continued until the endpoint. At study termination, mice were anesthetized and euthanized by overdose of isoflurane. Tumors and lungs were collected immediately and processed either by fixation in 4% PFA for downstream histology or snap-frozen and stored at −80 °C for later analysis.

### H&E histological staining

Hematoxylin and eosin (H&E) staining was performed according to our previously published methods.^67^ Briefly, paraffin-embedded tissues were sectioned at 5 μm, deparaffinized using Tissue-Clear (1466, Sakura), and rehydrated through a stepwise ethanol series (99%, 95%, and 70% ethanol). The tissue slides were stained with hematoxylin (6765009, Thermo Fisher Scientific), followed by eosin (HT110116, Sigma). After dehydration in 95% and 99% ethanol, the slides were mounted with PERTEX mounting medium (00801, HistoLab). The stained sections were imaged using a light microscope (20x, Nikon Eclipse TS100) equipped with a camera (DS-Fi1, Nikon) and analyzed with NIS-Elements F3.2 software.

### Immunohistochemistry

Immunohistochemical staining was carried out according to our previously published methods.^39^ Briefly, paraffin-embedded tissues were sectioned at 5 μm, deparaffinized in Tissue-Clear (1466, Sakura), and rehydrated through graded ethanol (99%, 95%, and 70%). Antigen retrieval was performed by boiling the sections for 20 min in unmasking solution (H3300, Vector). After blocking with 4% serum at room temperature, the sections were incubated overnight at 4 °C with primary antibodies against CD31 (1:100, 553370, BD Pharmingen), CD163 (1:100; 14-1631-82, Thermo Fisher Scientific), EpCAM (1:100; 14-5791-81, Thermo Fisher Scientific), CD86 (1:100, 19589, Cell Signaling), FAP (1:200, MAB9727, R&D Systems), α-SMA (1:200, MA5-11547, Thermo Fisher Scientific), desmin (1:200, MAB3430, Millipore), CA9 (1:300, NB100-417, Novus Biologicals), HIF1α (1:200, NB100-449, Novus Biologicals), F4/80 (1:200, 14-4801-82, Invitrogen), CD80 (1:200, 14-0081-82, Invitrogen), CD206 (1:200, 141702, Biolegend), CD3 (1:100, 17-0032-80, Thermo Fisher Scientific), CD4 (1:100, 550280, BD Pharmingen). Ready-to-use primary antibodies were also applied, including CD68 (M0876, Dako Agilent), CD163 (05973929001, Ventana), CD11 (M0717, Dako Agilent), CD20 (05267099001, Ventana), CD3 (NCL-L-CD3-565, Novocastra), CD4 (NCL-L-CD4-368, Novocastra), CD8 (M7103, Dako Agilent), MPO (05267692001, Ventana), Desmin (M7305, Dako Agilent), and α-SMA (M0851, Dako Agilent). After washing, the sections were incubated for 1 h at room temperature with secondary antibodies, including Alexa Fluor 488-labeled donkey anti-rat antibody (1:300; A21208, Thermo Fisher Scientific), Alexa Fluor 555 donkey anti-rabbit antibody (1:300; A31572, Thermo Fisher Scientific), Alexa Fluor 488 donkey anti-rabbit antibody (1:300; A21206, Thermo Fisher Scientific), Alexa Fluor 555 donkey anti-goat antibody (1:300; A21432, Thermo Fisher Scientific), Alexa Fluor 555 donkey anti-mouse antibody (1:300; A31570, Thermo Fisher Scientific), and Alexa Fluor 488 donkey anti-mouse antibody (1:300; A21202, Thermo Fisher Scientific). Fluorescent signals were acquired using a Nikon DS-Qi1MC fluorescence microscope, and images were processed using Adobe Photoshop CS6 (Adobe).

### Whole-mount staining

Whole-mount staining was performed according to previously described methods.^39^ Briefly, tissue samples were collected and fixed overnight in 4% PFA. Fixed specimens were sectioned and digested for 5 min with 20 mM proteinase K in 10 mM Tris-buffer (pH 7.5), followed by incubation in 100% methanol for 30 min. Sections were then incubated overnight at 4 °C in PBS containing 0.3% Triton X-100 and 3% skim milk. After five washes with PBS, samples were incubated overnight with a rat anti-mouse CD31 antibody (1:300, 553370, BD Pharmingen), followed by incubation with either an Alexa Fluor 488–labeled donkey anti-rat antibody (1:300, A21208, Thermo Fisher Scientific) or an Alexa Fluor 555–labeled donkey anti-rat antibody (1:300, A48270, Thermo Fisher Scientific). After the final PBS washes, the tissues were mounted in Vectashield medium (H1000, Vector Laboratories). Images were obtained by confocal microscopy (Nikon C1 Confocal microscope, Nikon Corporation, Japan). Dimensional images of vessels were analyzed. CD31-positive areas were quantified using Adobe Photoshop software (CS6, Adobe).

### Assessment of blood perfusion and vascular permeability

To evaluate vascular perfusion and leakage, mice received an intravenous injection via the tail vein of either 0.5 mg 2000 kDa lysinated rhodamine dextran (LRD; D7139, Thermo Fisher Scientific) or 0.6 mg 70 kDa LRD (D1818, Thermo Fisher Scientific). After circulation for 5 or 15 minutes, animals were euthanized by overdose of isoflurane. Lung tissues were harvested and fixed overnight in 4% paraformaldehyde. Samples were subsequently processed for whole-mount immunostaining following standard procedures. Endothelial structures were labeled using a rat anti-mouse CD31 antibody (1:200), followed by incubation with a Cy5-conjugated donkey anti-rat secondary antibody (1:200; Invitrogen). Fluorescent signals from LRD and CD31 staining were visualized using a confocal microscope (Nikon C1, Nikon Corporation), and three-dimensional image stacks were acquired. Vascular perfusion was quantified by measuring the area of the 2000 kDa LRD signal within vessels per imaging field, whereas vascular permeability was assessed by calculating the area of the extravascular 70 kDa LRD signal per field.

### RNA isolation and qPCR

RNA extraction and quantitative PCR were performed according to our previously published methods.^39^ Briefly, total RNA was extracted from tissues using TRIzol reagent (15-596-018, Invitrogen) and purified with the GeneJET RNA Purification Kit (K0731, Thermo Fisher Scientific) according to the manufacturers’ instructions. RNA concentrations were determined using a NanoDrop 2000 spectrophotometer. cDNA synthesis was performed with the RevertAid First Strand cDNA Synthesis Kit (K1622, Thermo Fisher Scientific), and the resulting cDNA was used for quantitative PCR (qPCR). qPCR was carried out using Power SYBR Green Master Mix (43-676-59, Invitrogen) on a StepOnePlus™ Real-Time PCR System. The relative gene expression levels were determined using the 2^−△△Ct^ method, with quantification based on the threshold cycle (Ct) values. The sequences of primers used in this study are provided in Suppl. Table 2.

### ELISA

Enzyme-linked immunosorbent assay (ELISA) was used to quantify VEGF and VEGF receptor protein levels according to the manufacturers’ protocols. Mouse VEGF concentrations were measured using the Mouse VEGF Quantikine ELISA Kit (MMV00, R&D Systems), and human VEGF concentrations were determined using the Human VEGF Quantikine ELISA Kit (DVE00, R&D Systems). Human VEGFR1 levels were measured using the Human VEGFR1/Flt-1 Quantikine ELISA Kit (DVR100C, R&D Systems), and human VEGFR2 levels were quantified using the Human VEGFR2/KDR Quantikine ELISA Kit (SVR200, R&D Systems). Absorbance was read at 450 nm using a microplate reader, and protein concentrations were calculated from standard curves using the corresponding trendline equations.

### Statistical analysis

Data analysis was conducted using GraphPad Prism (GraphPad Software, USA) and Microsoft Excel (Microsoft 365). The results are expressed as the mean ± standard error of the mean (s.e.m.). Statistical comparisons were performed using a two-tailed Student’s t-test and one-way analysis of variance (ANOVA). A *p*-value of < 0.05 was considered statistically significant.

## Data Availability

The source data supporting the findings of this study are provided in the Suppl. materials of this manuscript.

## Suppl. information

SUPPLEMENTAL MATERIAL_marked up
Dataset S1
