# Supplementary material for: COVID-19 triggers pulmonary tumor outgrowth through hypoxia- and inflammation-induced VEGF-dependent angiogenesis
Source: Signal Transduct Target Ther. 2026 Oct 12;11:441. doi: 10.1038/s41392-026-02952-6 (PMC13655177; doi:10.1038/s41392-026-02952-6)
Supplement: Supplementary file 1 [file 41392_2026_2952_MOESM1_ESM.docx]

Supplementary Materials for

COVID-19 triggers pulmonary tumor outgrowth through hypoxia- and

Inflammation-induced VEGF-dependent angiogenesis

Xu Jing, Mingwei Ma, Yan Bai, Yaping Wu, Xiaoting Sun, Jing Wu, Kristian Holgersson, Xiaolu Wang, Lijuan Chen, Siwen Long, Gerald M McInerney, Mikael Adner, Laszlo Szekely, Yunjian Zhang, Meiyun Wang, Jiaojiao Pang, Yuguo Chen and Yihai Cao

*Correspondence to: [yihai.cao@ki.se](mailto:yihai.cao@ki.se) or chen919085@sdu.edu.cn

**This PDF file includes:**

Figures. S1 to S17

Tables S1 to S2

**Other Supplementary Materials for this manuscript include the following:**

Data S1. Source and raw data for all experimental findings.

**Figure.S1**

**Supplementary Figure 1. SARS-CoV-2 infection augment inflammatory macrophages.**

Representative immunofluorescence staining of CD80 and CD206 (green) in lung sections from non-infected (NI) and SARS-CoV-2-infected mice, with nuclei counterstained by DAPI (blue). Quantification shows the percentage of CD80-positive and CD206-positive cells relative to total cells (n = 6-7 fields per group).

Data are presented as mean ± s.e.m. *p < 0.05, **p < 0.01, ***p < 0.001; two-sided unpaired t-test. Scale bars = 50 μm.

**Figure.S2**

**Supplementary Figure 2. Reduction of pulmonary T lymphocyte infiltration by Covid-19.**

Representative immunofluorescence staining of CD3, CD4, and CD8 (green) in lung sections from non-infected (NI) and SARS-CoV-2-infected mice, with nuclei counterstained by DAPI (blue). Quantification shows the percentage of CD3-positive, CD4-positive, and CD8-positive cells relative to total cells (n = 8 fields per group).

Data are presented as mean ± s.e.m. *p < 0.05, **p < 0.01, ***p < 0.001; two-sided unpaired t-test. Scale bars = 50 μm.

**Figure.S3**

**Supplementary Figure 3. SARS-CoV-2 infection triggers lung fibrosis.**

Representative immunofluorescence staining of FAP and αSMA (red) in lung sections from non-infected (NI) and SARS-CoV-2-infected mice, with nuclei counterstained by DAPI (blue). Quantification shows the FAP-positive area and αSMA-positive area (n = 8 fields per group).

Data are presented as mean ± s.e.m. *p < 0.05, **p < 0.01, ***p < 0.001; two-sided unpaired t-test. Scale bars = 50 μm.

**Figure.S4**

**Supplementary Figure 4. Different cell types of proliferation and apoptosis in SARS-CoV-2-infected lungs.**

**(a)** Representative immunofluorescence staining of TUNEL (red), Ki67 (red), Ki67 (red) and Desmin(green), Ki67 (red) and F4/80(green), Ki67 (red) and CD3(green), TUNEL (green) and Desmin (red), TUNEL (green) and F4/80 (red) and TUNEL (green) and CD31 (red) in lung sections from non-infected (NI) and SARS-CoV-2-infected mice, with nuclei counterstained by DAPI (blue). Quantification shows the percentage of Ki67-positive cells, TUNEL-positive cells, Ki67 and CD3 double-positive cells, Ki67 and Desmin double-positive cells, Ki67 and F4/80 double-positive cells, TUNEL and Desmin double-positive cells, TUNEL and F4/80 double-positive cells and TUNEL and CD31 double-positive cells relative to total cells (n = 8 fields per group).

**(b)** Relative mRNA expression levels of Vegfr1 in NI and SARS-Cov-2-infected mice (n = 8 samples per group).

Data are presented as mean ± s.e.m. *p < 0.05, **p < 0.01, ***p < 0.001; two-sided unpaired t-test. Scale bars = 50 μm.

**Figure.S5**

**Supplementary Figure 5. T cell apoptosis in SARS-CoV-2-infected lungs.**

Representative immunofluorescence staining of TUNEL (green) and CD3 (red), TUNEL (green) and CD4 (red) and TUNEL (green) and CD8 (red) in lung sections from non-infected (NI) and SARS-CoV-2-infected mice, with nuclei counterstained by DAPI (blue). Quantification shows the percentage of TUNEL and CD3 double-positive cells, TUNEL and CD4 double-positive cells and TUNEL and CD8 double-positive cells relative to total cells (n = 8 fields per group).

Data are presented as mean ± s.e.m. *p < 0.05, **p < 0.01, ***p < 0.001; two-sided unpaired t-test. Scale bars = 50 μm.

**Figure.S6**

**Supplementary Figure 6. Tumor hypoxia, proliferation and apoptosis in tumor-bearing Covid-19 mice.**

**(a)** Circulating VEGF (cVEGF) concentration in plasma from non-infected (NI) and SARS-CoV-2-infected (SC) mice measured by ELISA (n = 7 mice per group).

**(b)** Representative immunofluorescence staining of HIF1α (red) and CA9 (red) in lung tumors from SARS-CoV-2-infected mice, with nuclei counterstained by DAPI (blue). Quantification of HIF1α-positive area and CA9-positive area in tumors from non-infected (NI) and SARS-CoV-2-infected (SC) mice (n = 8 fields per group). Representative immunofluorescence staining of Ki67 (red) and TUNEL (red) in lung tumors, with quantification of the percentage of Ki67-positive cells and TUNEL-positive cells relative to total cells (n = 8 fields per group).

Data are presented as mean ± s.e.m. *p < 0.05, **p < 0.01, ***p < 0.001; two-sided unpaired t-test. Scale bars = 50 μm.

**Figure.S7**

**Supplementary Figure 7. Tumor T lymphocytes in tumor-bearing Covid-19 mice.**

Representative immunofluorescence staining of CD3, CD4, and CD8 (green) in lung tumor sections from non-infected (NI) and SARS-CoV-2-infected mice, with nuclei counterstained by DAPI (blue). Quantification shows the percentage of CD3-positive, CD4-positive, and CD8-positive cells relative to total cells in tumor regions (n = 8 fields per group).

Data are presented as mean ± s.e.m. *p < 0.05, **p < 0.01, ***p < 0.001; two-sided unpaired t-test. Scale bars = 50 μm.

**Figure.S8**

**Supplementary Figure 8. Tumor macrophages in tumor-bearing Covid-19 mice.**

Representative immunofluorescence staining of F4/80 (green), CD80 (green) and CD86 (Red), CD163 (Red), CD206 (green) in lung tumor sections from non-infected (NI) and SARS-CoV-2-infected mice, with nuclei counterstained by DAPI (blue). Quantification shows the percentage of F4/80-positive, CD80-positive, CD86-positive, CD163-positive and CD206-positive cells relative to total cells in tumor regions (n = 8 fields per group).

Data are presented as mean ± s.e.m. *p < 0.05, **p < 0.01, ***p < 0.001; two-sided unpaired t-test. Scale bars = 50 μm.

**Figure.S9**

**Supplementary Figure 9. Tumor fibroblasts in tumor-bearing Covid-19 mice.**

Representative immunofluorescence staining of FAP, Desmin, and αSMA (red) in lung tumor sections from non-infected (NI) and SARS-CoV-2-infected mice, with nuclei counterstained by DAPI (blue). Quantification shows the FAP-positive area, Desmin-positive area, and αSMA-positive area (n = 8 fields per group).

Data are presented as mean ± s.e.m. *p < 0.05, **p < 0.01, ***p < 0.001; two-sided unpaired t-test. Scale bars = 50 μm.

**Figure.S10**

**Supplementary Figure 10. Cell proliferation and apoptosis in the anti-VEGF-treated SARS-CoV-2-infected lungs.**

**(a)** Total VEGF production in 2 x 10^5^ LLC-vec and LLC-VEGF cell line measured by ELISA (n = 8 samples per group).

**(b)** Representative immunofluorescence staining of Ki67 (red) and TUNEL (red) in lung sections from NIIgG- and anti-VEGF-treated NI- or SARS-CoV-2-infected mice, with nuclei counterstained by DAPI (blue). Quantification shows the percentage of Ki67-positive cells and TUNEL-positive cells relative to total cells (n =7-8 fields per group).

Data are presented as mean ± s.e.m. *p < 0.05, **p < 0.01, ***p < 0.001; one-way ANOVA with Tukey's post-hoc test. NS = not significant. Scale bars = 50 μm.

**Figure.S11**

**Supplementary Figure 11. Tumor cell proliferation and apoptosis in the anti-VEGF-treated tumor-bearing Covid-19 mice.**

Representative immunofluorescence staining of Ki67 (red) and TUNEL (red) in lung tumor sections from NIIgG- and anti-VEGF-treated NI- or SARS-CoV-2-infected mice, with nuclei counterstained by DAPI (blue). Quantification shows the percentage of Ki67-positive cells and TUNEL-positive cells relative to total cells in tumor regions (n = 8 fields per group).

Data are presented as mean ± s.e.m. *p < 0.05, **p < 0.01, ***p < 0.001; one-way ANOVA with Tukey's post-hoc test. NS = not significant. Scale bars = 50 μm.

**Figure.S12**

**Supplementary Figure 12. T lymphocytes in the anti-VEGF-treated SARS-CoV-2-infected lungs.**

Representative immunofluorescence staining of CD3, CD4, and CD8 (green) in lung sections from NIIgG- and anti-VEGF-treated NI- or SARS-CoV-2-infected mice, with nuclei counterstained by DAPI (blue). Quantification shows the percentage of CD3-positive, CD4-positive, and CD8-positive cells relative to total cells (n = 8 fields per group).

Data are presented as mean ± s.e.m. *p < 0.05, **p < 0.01, ***p < 0.001; one-way ANOVA with Tukey's post-hoc test. NS = not significant. Scale bars = 50 μm.

**Figure.S13**

**Supplementary Figure 13. Tumor T lymphocytes in the anti-VEGF-treated tumor-bearing Covid-19 mice.**

Representative immunofluorescence staining of CD3, CD4, and CD8 (green) in lung tumor sections from NIIgG- and anti-VEGF-treated NI- or SARS-CoV-2-infected mice, with nuclei counterstained by DAPI (blue). Quantification shows the percentage of CD3-positive, CD4-positive, and CD8-positive cells relative to total cells in tumor regions (n = 8 fields per group).

Data are presented as mean ± s.e.m. *p < 0.05, **p < 0.01, ***p < 0.001; one-way ANOVA with Tukey's post-hoc test. NS = not significant. Scale bars = 50 μm.

**Figure.S14**

**Supplementary Figure 14. Macrophages in the anti-VEGF-treated SARS-CoV-2-infected lungs.**

Representative immunofluorescence staining of F4/80, CD206, and CD80 (green) in lung sections from NIIgG- and anti-VEGF-treated NI- or SARS-CoV-2-infected mice, with nuclei counterstained by DAPI (blue). Quantification shows the percentage of F4/80-positive, CD206-positive, and CD80-positive cells relative to total cells (n = 8 fields per group).

Data are presented as mean ± s.e.m. *p < 0.05, **p < 0.01, ***p < 0.001; one-way ANOVA with Tukey's post-hoc test. NS = not significant. Scale bars = 50 μm.

**Figure.S15**

**Supplementary Figure 15. Tumor macrophages in the anti-VEGF-treated tumor-bearing Covid-19 mice.**

Representative immunofluorescence staining of F4/80, CD206, and CD80 (green) in lung tumor sections from NIIgG- and anti-VEGF-treated NI- or SARS-CoV-2-infected mice, with nuclei counterstained by DAPI (blue). Quantification shows the percentage of F4/80-positive, CD206-positive, and CD80-positive cells relative to total cells in tumor regions (n = 8 fields per group).

Data are presented as mean ± s.e.m. *p < 0.05, **p < 0.01, ***p < 0.001; one-way ANOVA with Tukey's post-hoc test. NS = not significant. Scale bars = 50 μm.

**Figure.S16**

**Supplementary Figure 16. Fibroblasts in the anti-VEGF-treated SARS-CoV-2-infected lungs.**

Representative immunofluorescence staining of FAP, Desmin, and αSMA (red) in lung sections from NIIgG- and anti-VEGF-treated NI- or SC-infected mice, with nuclei counterstained by DAPI (blue). Quantification shows the FAP-positive area, Desmin-positive area, and αSMA-positive area (n = 8 - 9 fields per group).

Data are presented as mean ± s.e.m. *p < 0.05, **p < 0.01, ***p < 0.001; one-way ANOVA with Tukey's post-hoc test. NS = not significant. Scale bars = 50 μm.

**Figure.S17**

**Supplementary Figure 17. Tumor fibroblasts in the anti-VEGF-treated tumor-bearing Covid-19 mice.**

Representative immunofluorescence staining of FAP, Desmin, and αSMA (red) in lung tumor sections from NIIgG- and anti-VEGF-treated NI- or SARS-CoV-2-infected mice, with nuclei counterstained by DAPI (blue). Quantification shows the FAP-positive area, Desmin-positive area, and αSMA-positive area (n = 8 fields per group).

Data are presented as mean ± s.e.m. *p < 0.05, **p < 0.01, ***p < 0.001; one-way ANOVA with Tukey's post-hoc test. NS = not significant. Scale bars = 50 μm.

**Supplementary Table 1.** Clinical characteristics

| **Groups** | **Age (years)** | **Sex** | **Death cause** | **Lung weight (g)** |
| --- | --- | --- | --- | --- |
| **Non-infected** |  |  |  |  |
| Patient 1 | 75 | Female | Septic shock | 1206 |
| Patient 2 | 71 | Female | Acute necrotizing esophagitis | 988 |
| Patient 3 | 74 | Female | Septic shock secondary to postoperative infection | 961 |
| Patient 4 | 71 | Female | Paralytic ileus/Acute circulatory failure | 839 |
| Patient 5 | 59 | Male | Food bolus obstruction with aspiration | 1768 |
| Patient 6 | 82 | Female | Acute circulatory failure | 704 |
| Patient 7 | 42 | Male | Plasmacytoid leukemia | 1890 |
| Patient 8 | 74 | Female | Acute myocardial infarction | 1080 |
| Patient 9 | 58 | Female | Cardiac failure | 1454 |
| Patient 10 | 35 | Male | Acute hemorrhagic necrotizing pneumonia | 1322 |
| Patient 11 | 69 | Male | Acute circulatory failure | 1374 |
| Patient 12 | 53 | Male | Septic shock | 1815 |
| Patient 13 | 46 | Male | Sepsis secondary to tongue root abscess | 1473 |
| Patient 14 | 74 | Female | Acute myocardial infarction | 868 |
| Patient 15 | 83 | Female | Ruptured aortic dissection causing cardiac tamponade | 1115 |
| **Covid-19** |  |  |  |  |
| Patient 1 | 56 | Female | Severe Covid-19 | 2710 |
| Patient 2 | 39 | Female | Severe Covid-19 | 1980 |
| Patient 3 | 23 | Female | Severe Covid-19 | 1630 |
| Patient 4 | 60 | Male | Severe Covid-19 | 2260 |
| Patient 5 | 81 | Female | Severe Covid-19 | 1260 |
| Patient 6 | 39 | Female | Severe Covid-19 | 1346 |
| Patient 7 | 68 | Male | Severe Covid-19 | 1601 |
| Patient 8 | 66 | Male | Severe Covid-19 | 1743 |
| Patient 9 | 69 | Male | Severe Covid-19 | 1227 |
| Patient 10 | 61 | Male | Severe Covid-19 | 2732 |
| Patient 11 | 74 | Female | Severe Covid-19 | 1489 |
| Patient 12 | 56 | Male | Severe Covid-19 | 2057 |
| Patient 13 | 56 | Male | Severe Covid-19 | 1536 |
| Patient 14 | 81 | Male | Severe Covid-19 | 1309 |
| Patient 15 | 64 | Male | Severe Covid-19 | 1469 |
| Patient 16 | 57 | Male | Severe Covid-19 | 1045 |
| Patient 17 | 65 | Male | Severe Covid-19 | 3654 |
| Patient 18 | 77 | Male | Severe Covid-19 | 2379 |
| Patient 19 | 58 | Male | Severe Covid-19 | 1595 |
| Patient 20 | 68 | Male | Severe Covid-19 | 1250 |
| Patient 21 | 56 | Male | Severe Covid-19 | 2370 |

**Supplementary Table 2.** Primer sequences

| Gene | Species | Forward primer sequence | Reverse primer sequence |
| --- | --- | --- | --- |
| *Actin* | mouse | 5’-AGGCCCAGAGCAAGAGAGG-3’ | 5’-TACATGGCTGGGGTGTTGAA-3’ |
| *Car9* | mouse | 5’-CTGAAGACAGGATGGAGAAG-3’ | 5’-GCAGAGTGCGGCAGAATG-3’ |
| *Hif1α* | mouse | 5’-GTCGGACAGCCTCACCAAACAG-3’ | 5’-TAGGTAGTGAGCCACCAGTGTCC-3’ |
| *Vegf* | mouse | 5’-ATCCGCATGATCTGCATGG-3’ | 5’-AGTCCCATGAAGTGATCAAGTTCA-3’ |
| *Vegfr2* | mouse | 5’- CTCTGTGGGTTTGCCTGGCGATTTTCT -3’ | 5’- GGGGATCACCACAGTTTTGTTCTTGTT -3’ |
| *Vegfr1* | mouse | 5’- AGCCCACCTCTCTATCCGCTGG -3’ | 5’- GGCGCTTCCGAATCTCTAACG -3’ |
| *ACTIN* | human | 5’-ATTGCCGACAGGATGCAGAA-3’ | 5’-GCTGATCCACATCTGCTGGAA-3’ |
| *CA9* | human | 5’- GGAAGGCTCAGAGACTCA -3’ | 5’- CTTAGCACTCAGCATCAC -3’ |
| *HIF1α* | human | 5’- TCACCTGAGCCTAATAGTCC -3’ | 5’- GCTAACATCTCCAAGTCTAA-3’ |
| *VEGF* | human | 5’- TGCAGATTATGCGGATCAAACC -3’ | 5’- TGCATTCACATTTGTTGTGCTGTAG -3’ |
| *PECAM* | human | 5’- AACAGTGTTGACATGAAGAGCC -3’ | 5’- TGTAAAACAGCACGTCATCCTT -3’ |
| *VEGFR2* | human | 5’- CCAGCAAAAGCAGGGAGTCTGT -3’ | 5’- TGTCTGTGTCATCGGAGTGATATCC -3’ |
| *VEGFR1* | human | 5’- CAGGCCCAGTTTCTGCCATT -3’ | 5’- TTCCAGCTCAGCGTGGTCGTA -3’ |

**Data S1.** Source and raw data for all experimental findings.
